# Exaggerated hypoxic vascular breakdown in aged brain due to reduced microglial vasculo‐protection

**DOI:** 10.1111/acel.13720

**Published:** 2022-09-21

**Authors:** Sebok K. Halder, Richard Milner

**Affiliations:** ^1^ San Diego Biomedical Research Institute San Diego California USA

**Keywords:** aging, angiogenesis, blood vessels, blood‐brain barrier integrity, brain, chronic mild hypoxia, endothelial proliferation, fibrinogen, microglia

## Abstract

In a recent study of young mice, we showed that chronic mild hypoxia (CMH, 8% O_2_) triggers transient blood–brain barrier (BBB) disruption, and that microglia play an important vasculo‐protective function in maintaining BBB integrity. As hypoxia is a common component of many age‐related diseases, here we extended these studies to aged mice and found that hypoxia‐induced vascular leak was greatly amplified (5‐fold to 10‐fold) in aged mice, being particularly high in the olfactory bulb and midbrain. While aged mice showed no obvious difference in the early stages of hypoxic angiogenic remodeling, the compensatory increase in vascularity and vessel maturation was significantly delayed. Compared with young brain, microglia in the normoxic aged brain were markedly activated, and this was further increased under hypoxic conditions, but paradoxically, this correlated with reduced vasculo‐protection. Microglial depletion studies showed that microglial still play an important vasculo‐protective role in aged brain, but interestingly, partial attenuation of microglial activation with minocycline resulted in fewer vascular leaks and reduced loss of endothelial tight junction proteins. Taken together, these findings suggest that increased BBB disruption in hypoxic aged mice can be explained both by a delayed vascular remodeling response and reduced microglial vasculo‐protection. Importantly, they show that overly activated microglia in the aged brain are less effective at maintaining vascular integrity, though this can be improved by reducing microglial activation with minocycline, suggesting therapeutic potential for enhancing BBB integrity in the hypoxia‐predisposed elderly population.

## Graphical abstract

Hypoxia leads to blood–brain barrier disruption and extravascular leak of fibrinogen. In young mice, this promotes microglial activation and migration and aggregation around the site of vascular leak, leading to vasculo‐protection. In aged mice, despite being more activated, microglia migrate much slower to sites of vascular breakdown, resulting in much greater levels of vascular leak. Attenuation of microglial activation with minocycline enhances vasculo‐protection, resulting in decreased levels of vascular leak.

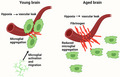

## 1 INTRODUCTION

Blood vessels in the central nervous system (CNS) have uniquely high electrical resistance and low permeability, which creates a very selective barrier separating the blood and CNS parenchymal tissue. This blood–brain barrier (BBB) protects sensitive neural cells from potentially harmful components in the blood (Ballabh et al., 2004; Huber et al., 2001) and allows transport of only those metabolites (e.g., glucose and amino acids) that CNS tissue specifically needs. The basis of the BBB depends on a combination of structures, including endothelial adherens and tight junction protein complexes, extracellular matrix (ECM) components of the vascular basal lamina, and the influence of neighboring CNS‐resident cells including astrocytes and pericytes (Daneman et al., 2010; del Zoppo & Milner, 2006; Roberts et al., 2015). BBB breakdown occurs in many neurological conditions, including meningitis, ischemic stroke, multiple sclerosis (MS), and CNS tumors (Davies, 2002; Gay & Esiri, 1991; Roberts et al., 2015). Recent studies suggest that BBB integrity also deteriorates as part of the normal aging process and in vascular dementia (Farrall & Wardlaw, 2009; Senatorov et al., 2019), also known as vascular contributions to cognitive impairment and dementia (VCID), which by upsetting the tightly controlled CNS milieu, predisposes to neuronal dysfunction and neurodegeneration (Banks et al., 2021; Levit et al., 2020).

BBB integrity is also disrupted by low oxygen levels (hypoxia) (Bauer et al., 2010; Schoch et al., 2002). In humans, this has been well documented in mountaineers who when exposed to decreasing oxygen concentration at higher altitudes, can present with acute mountain sickness (AMS), consisting of confusion and headaches. If left untreated, this can evolve into life‐threatening high‐altitude cerebral edema (HACE), which necessitates immediate evacuation to a lower altitude, where the patient quickly recovers (Davis & Hackett, 2017). When rodents are exposed to chronic mild hypoxia (CMH, typically 8%–10% O_2_), cerebral blood vessels mount a strong adaptive remodeling response, resulting in 50% increased vessel density over a period of 2–3 weeks (LaManna et al., 2004) in order to maintain the brain's metabolic demands.

Recently, we demonstrated that CMH promotes transient vascular leak in cerebral blood vessels in young C57BL6/J mice, that is associated with aggregation and activation of microglia around disrupted vessels (Halder & Milner, 2020). Interestingly, microglial depletion profoundly increases hypoxia‐induced cerebrovascular leak, suggesting an important vasculo‐protective role for microglia under hypoxic conditions. As an intact BBB is a vital prerequisite for the maintenance of cerebral health, yet evidence suggests that BBB integrity declines with age (Farrall & Wardlaw, 2009; Senatorov et al., 2019), the aim of this study was to examine how these events are influenced by aging. This is important because the risk of hypoxia is far greater in the aged due to declining pulmonary, cardiac, and cerebrovascular function, which could predispose to BBB disruption, neurodegeneration, and cognitive decline. This concept also has strong clinical relevance to the often‐severe level of hypoxia reported in SARS‐CoV‐2 patients (Bhatia & Mohammed, 2020). Based on this, the goal of this study was to address the following questions: (i) how does aging affect hypoxia‐induced cerebrovascular leak, (ii) which regions of the brain are affected, (iii), is the vasculo‐protective function of microglia affected by age, and (iv) if it is, can manipulation of microglial activation state improve vasculo‐protective function in the aged brain?

## 2 RESULTS

### 2.1 Aged mice show greatly enhanced cerebrovascular leak in response to hypoxia

**FIGURE 1:**
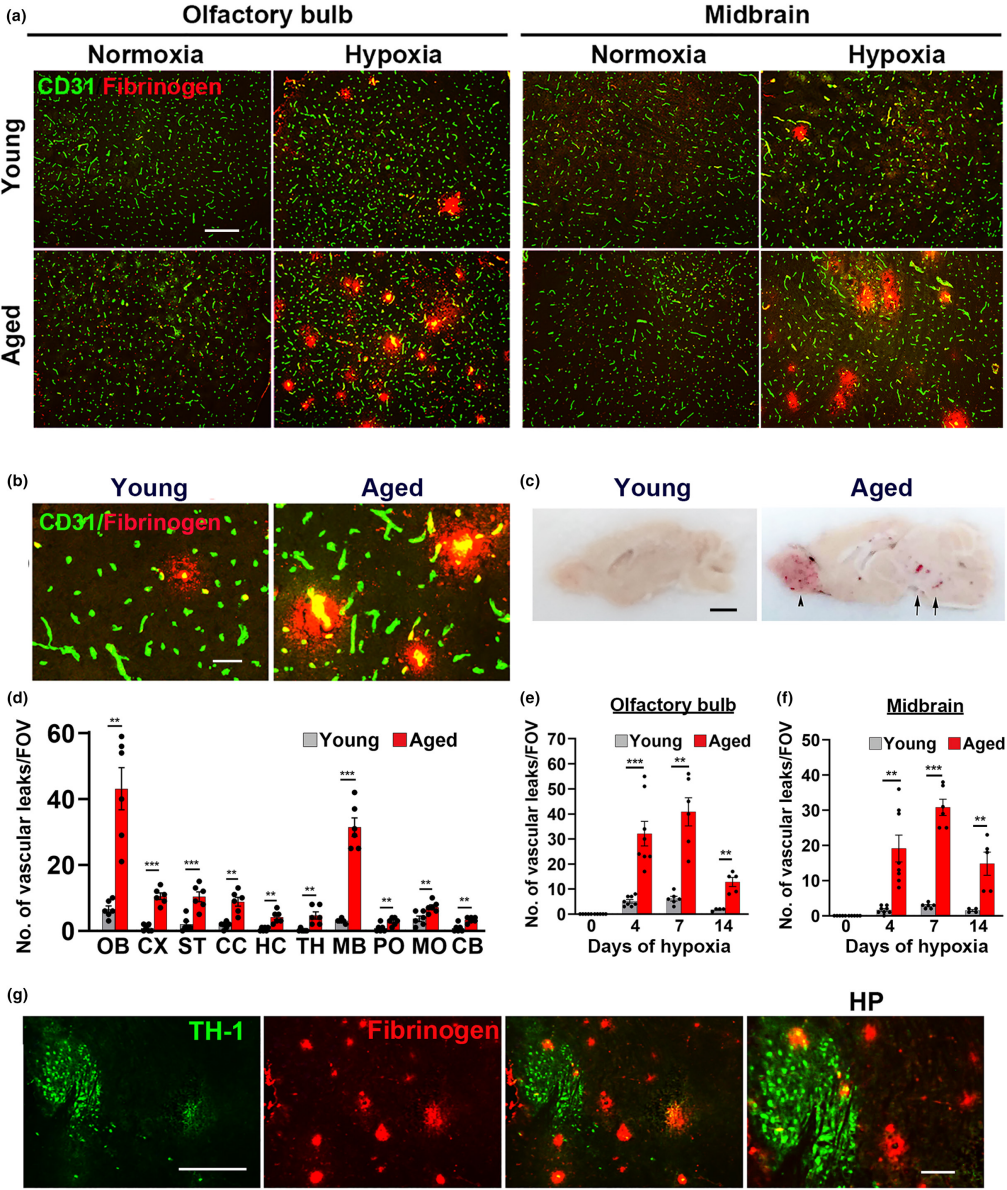
Chronic mild hypoxia (CMH)‐induced vascular leak is much greater in aged brain. (a) Frozen brain sections taken from young (8–10 weeks) or aged (20 months) mice exposed to normoxia or 7‐day hypoxia (8% O_2_) were stained for the endothelial marker CD31 (AlexaFluor‐488) and fibrinogen (Cy‐3). Images were captured in the olfactory bulb and the midbrain. Scale bar = 200 μm. (b) High power image of olfactory bulb shown in A. Scale bar = 50 μm. (c) Comparison of young and aged brains at time of sectioning. Scale bar = 2 mm. (d) Quantification of the number of vascular leaks (fibrinogen‐positive)/FOV in different brain regions after 7‐day hypoxia. OB, olfactory bulb; CX, cerebral cortex; ST, striatum; CC, corpus callosum; HC, hippocampus; TH, thalamus; MB, midbrain; PO, pons; MO, medulla oblongata; CB, cerebellum. (e,f) Quantification of the number of vascular leaks in the olfactory bulb and midbrain after 0‐, 4‐, 7‐, and 14‐day hypoxia. All results are expressed as the mean ± SEM (*n* = 4–8 mice/group). **p < 0.01, ***p < 0.001 vs. normoxic conditions. Note that vascular leak was much greater in aged brain and that the highest number of vascular leaks occurred in the olfactory bulb and midbrain, peaking after 7‐day hypoxia but declined by Day 14. (g) Frozen brain sections taken from mice exposed to 7‐day hypoxia (8% O_2_) were dual‐labeled for tyrosine hydroxylase‐1 (TH‐1) (AlexaFluor‐488) and fibrinogen (Cy‐3). Scale bar = 200 μm except for high power (HP) image on the right, where scale bar = 50 μm. Note that hypoxia‐induced vascular leak occurred in the TH‐1+ substantia nigra (SN) and in the surrounding red nucleus.

The extent of cerebrovascular leak in young (8–10 weeks) and aged (20 months) female C57BL6/J mice was compared after exposure to chronic mild hypoxia (CMH, 8% O_2_) for periods up to 14 days, by dual‐immunofluorescence (dual‐IF) of frozen brain sections, using CD31 to label endothelial cells and fibrinogen to identify extravascular leak. As shown in Figure 1a,b, while no vascular leak occurred in young or aged brains under normoxic conditions, CMH triggered extravascular leak in a small number of cerebral blood vessels in young brains, but in aged brains, this number was greatly increased. Interestingly, hypoxia‐induced vascular breakdown was so obvious in aged brains it was first evident at time of tissue harvesting, when cerebral hemorrhage was observed in the olfactory bulb and midbrain of hypoxic aged mice, something never seen in brains of hypoxic young mice (Figure 1c).

### 2.2 Hypoxia‐induced vascular leak in aged brain occurs predominantly in the olfactory bulb and midbrain

We next quantified the density of vascular leaks in 10 different brain regions (Figure 1d). Consistent with our prior study, this showed that in young brain, the olfactory bulb (OB) and the medulla oblongata (MO) contained the highest number of vascular leaks. In the aged brain, the olfactory bulb also contained the highest density of vascular leaks, but the midbrain (MB) also stood out in showing a much greater number of leaks than other regions (Figure 1d; *p* < 0.01). Quantification revealed that the number of vascular leaks/field of view (FOV) (Figure 1e,f) or the area of vascular leaks/FOV (Figure S1) in aged brains was far greater than young brains in all regions examined. At both ages, vascular leak was highest between 4 and 7 days of CMH but declined at the 14‐day timepoint. This makes the important point that even in aged brain, where much greater vascular leak occurs, cerebral blood vessels still retain an inherent capacity to repair and resolve vascular leaks, despite the continued presence of hypoxia. As vascular leak also leads to proteolytic degradation of endothelial tight junction proteins (Boroujerdi et al., 2015; Halder & Milner, 2019), we next examined how this differs between young and aged brains. Compared to the young brain, blood vessels in the aged brain showed much greater loss of both ZO‐1 and occludin (*p* < 0.01; Figure S2).

Closer analysis of the midbrain revealed that vascular leaks were detected across a widespread area, including the substantia nigra (SN), as detected by tyrosine hydroxylase‐1 (TH‐1)‐positive neurons (Figure 1g), and in the red nucleus (area outlined in Figure S3a). In the olfactory bulb, vascular leaks were distributed both in the NeuN‐rich gray matter and adjacent white matter (Figure S3b). Vascular leaks also occurred in the corpus callosum and cerebral cortex and were generally larger in the corpus callosum (white matter) compared to the cerebral cortex (gray matter) (Figure S3c). In summary, these studies show that CMH provokes much greater vascular leak in the aged brain, particularly in the midbrain and olfactory bulb. Based on these findings, we focussed our subsequent analysis in these regions.

Because the olfactory bulb was the area of brain showing the greatest extent of vascular breakdown, we next tested whether the sense of smell (olfactory function) was altered in mice exposed to hypoxia using the well‐established buried food test. This test evaluates the ability of mice to locate a food source, in this case a small cookie, hidden beneath a layer of bedding. Under normoxic conditions, this revealed that aged mice took twice as long as young mice to find the hidden food (*p* < 0.05; Figure S4). Even more striking was the finding that mice exposed to 4‐day CMH (both young and aged) took much longer to find the hidden food (*p* < 0.001), suggesting that olfactory function is markedly compromised following hypoxic exposure.

### 2.3 Hypoxia‐induced cerebrovascular remodeling is delayed in aged mice

**FIGURE 2:**
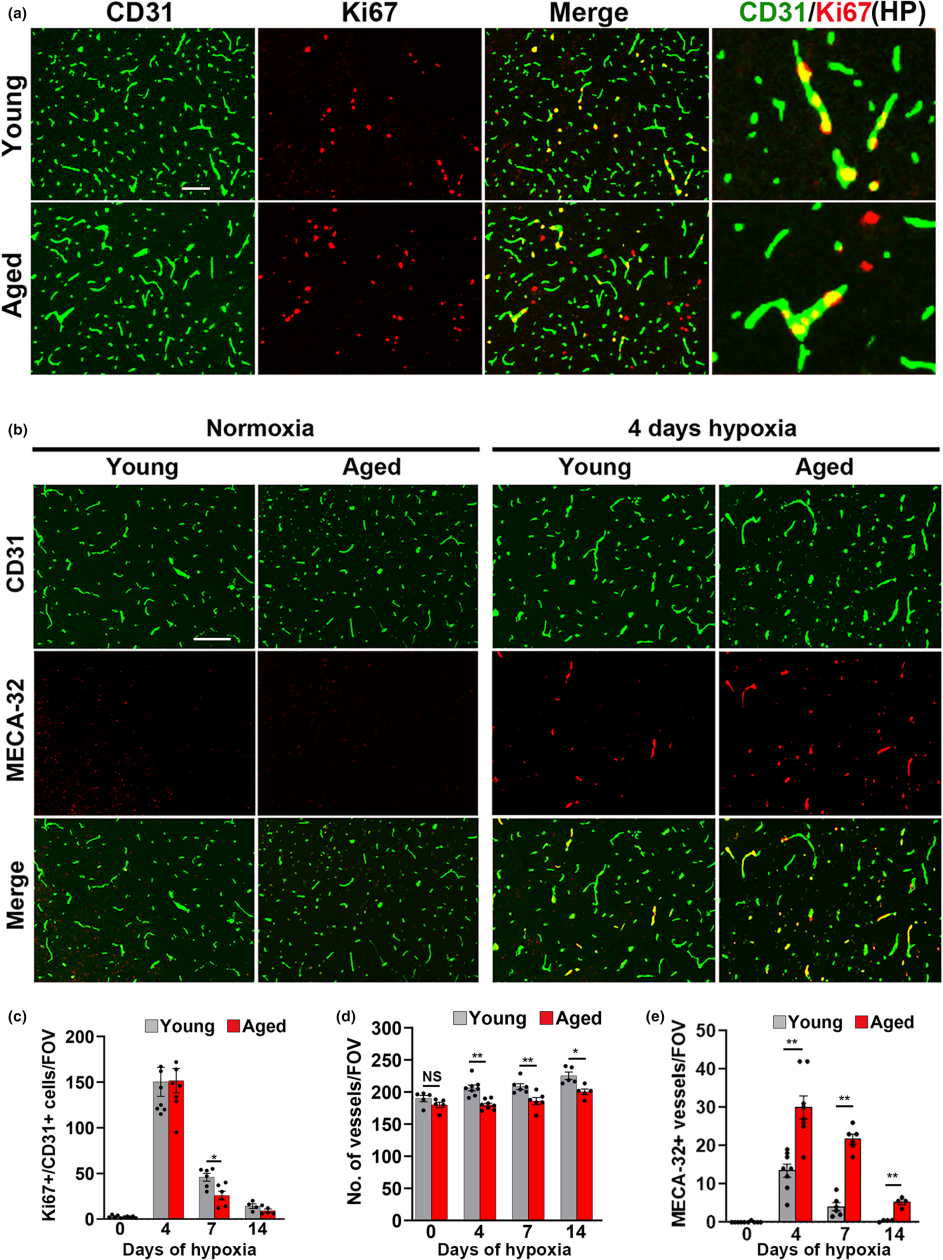
Evaluation of cerebrovascular remodeling in young and aged mice exposed to hypoxia. (a) Frozen brain sections (images show midbrain) taken from young (8–10 weeks) or aged (20 months) mice exposed to hypoxia (8% O_2_) for 4 days were stained for CD31 (AlexaFluor‐488) and the proliferation marker Ki67 (Cy‐3). Scale bar = 100 μm. High power (HP) images on the right highlight CD31/Ki67 co‐localization. (b) CD31/MECA‐32 dual‐IF of frozen brain sections (midbrain shown) taken from mice exposed to normoxia or 4‐day hypoxia. Scale bar = 100 μm. (c–e) Quantification of the number of proliferating endothelial cells (CD31^+^/Ki67^+^ cells)/FOV (c), number of blood vessels/FOV (d), and number of MECA‐32+ vessels/FOV (e) after 0‐, 4‐, 7‐, and 14‐day hypoxia. Results are expressed as the mean ± SEM (*n* = 4–8 mice/group). *p < 0.05, **p < 0.01. Note that the aged brain showed a faster drop‐off of hypoxia‐induced endothelial proliferation, attenuation of increased vascularity response, and a greater number of MECA‐32+ vessels at all time‐points.

Our studies raise the obvious question: why is hypoxia‐induced cerebrovascular leak so much greater in the aged? Is it due to age‐related changes in the inherent properties of endothelial cells, or alternatively, based on our recent finding of a critical vasculo‐protective role of microglia (Halder & Milner, 2019, 2020), is it the result of declining vasculo‐protective microglial function in the aged? To answer this question, we first compared CMH‐induced vascular remodeling responses in the midbrain of young and aged mice, using CD31/Ki67 dual‐IF to quantify endothelial proliferation, a key early step in the angiogenic process. This revealed that in the brains of both ages, CMH triggered a robust endothelial proliferation response (Figure 2a and Figures S5 and S6) which peaked after 4‐day CMH, and slowly declined at later timepoints (Figure 2c). Interestingly, while there was no age‐related difference in the endothelial proliferation rate at the 4‐day timepoint, at later timepoints, aged brains showed a more rapid fall‐off in endothelial proliferation (Day 7, *p* < 0.05).

As the end‐product of CMH‐induced vascular remodeling is enhanced vascularity (LaManna, Chavez, & Pichiule, 2004; LaManna, Vendel, & Farrell, 1992), we next examined how 14‐day CMH influences blood vessel density in the midbrain. This showed that although CMH induced significant increases in vessel density in the midbrain of both young and aged mice, the changes in aged brains were significantly attenuated compared to young brains (*p* < 0.01 at Days 4 and 7, *p* < 0.05 at Day 14; Figure 2d). This suggests that while the angiogenic response starts off equally fast in young and aged brains, the more rapid fall‐off in endothelial proliferation in aged brains results in delayed vascularization. To seek confirmation of this, we compared young and aged brains for expression of MECA‐32, a marker of immature/remodeling cerebral blood vessels (Engelhardt et al., 1994; Hallman et al., 1995). As expected, no MECA‐32 expression was detected under normoxic conditions in young or aged brains (Figure 2b,e). However, following CMH exposure, aged mice showed a greatly increased number of MECA‐32‐positive blood vessels compared to young mice at all timepoints examined (*p* < 0.01, Figure 2b,e). In parallel, the expression of the endothelial activation marker vascular cell adhesion molecule (VCAM)‐1 was also significantly higher in aged mice compared to young mice at all hypoxic timepoints examined (Figure S7; *p* < 0.001). These data support the concept that delayed blood vessel maturation in aged mice leads to extended times of vascular vulnerability and greater subsequent leak. They also imply that the greater hypoxic vulnerability of aged cerebral blood vessels is due, at least in part, to intrinsic age‐related changes in endothelial properties. In our previous studies in young mice, we described CMH‐induced astrocyte‐vascular uncoupling as shown by vascular loss of aquaporin‐4 (AQP4) expression, the marker of astrocyte endfeet (Halder & Milner, 2019, 2020). To examine how aging impacts this uncoupling, we performed CD31/AQP4/fibrinogen triple‐IF and this revealed in both young and aged brain, that within regions of extravascular fibrinogen leak, a significant % of blood vessels had lost AQP4 expression (Figure S8). Interestingly, while there was a greater number of AQP4‐negative blood vessels within the hypoxic aged brain (not unexpected because of the greater number of leaks in aged mice), the % of vessels within leaky areas that were AQP4‐negative was not noticeably different between the two age groups.

### 2.4 Microglial activation is enhanced by age and CMH, but microglial vasculo‐protection declines with age

**FIGURE 3:**
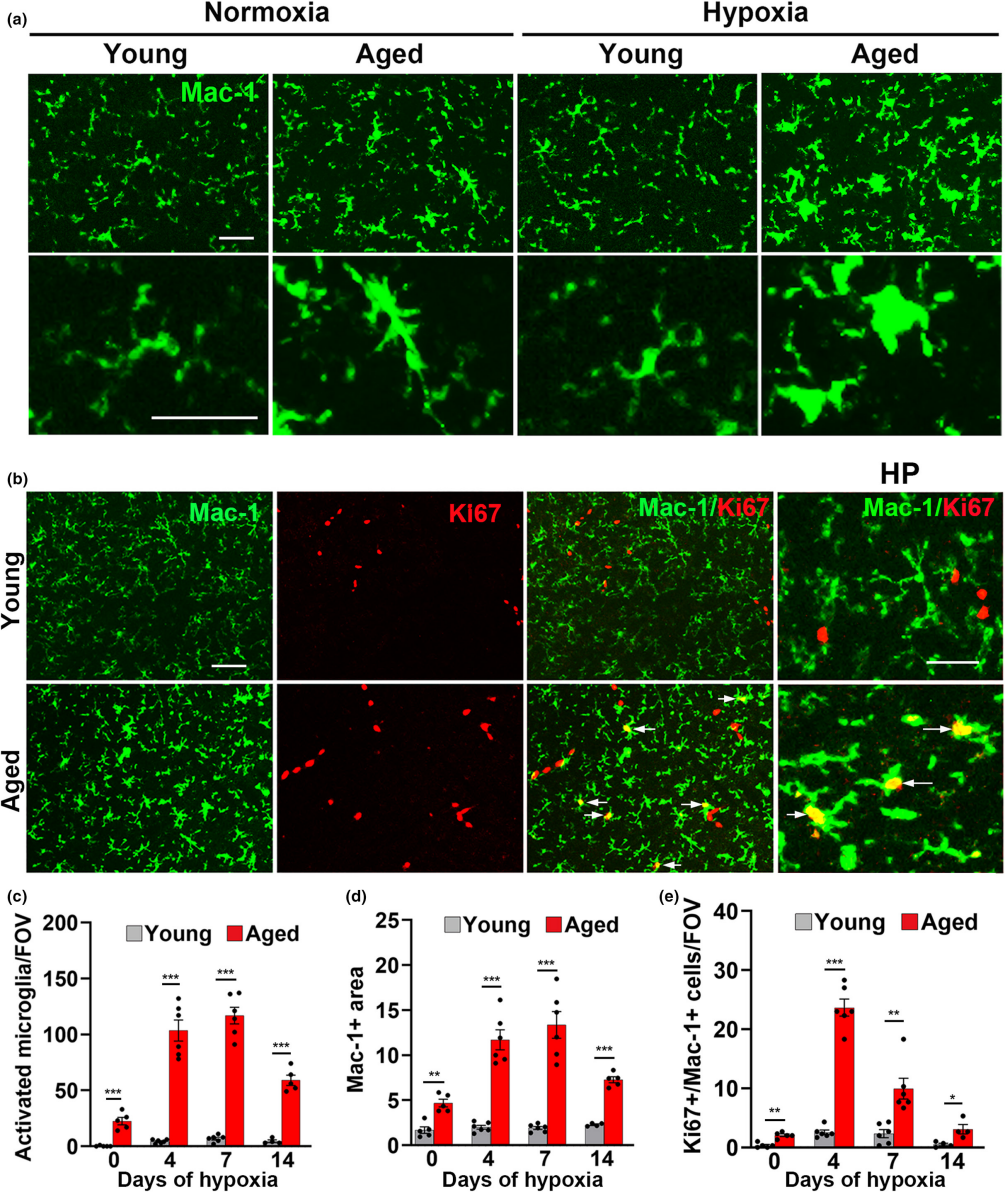
Microglia in aged brain show much greater activation. Frozen brain sections taken from young (8–10 weeks) or aged (20 months) mice exposed to normoxia or hypoxia (8% O_2_) for 4 days were stained for Mac‐1 (AlexaFluor‐488) (a) or Mac‐1 (AlexaFluor‐488) and the proliferation marker Ki67 (Cy‐3) (b). Images were captured in the midbrain. Scale bars = 50 μm (a) or 100 μm (b). Quantification of the number of morphologically activated microglia/FOV (c), total Mac‐1 area/FOV (d) and number of Ki67+/Mac‐1+ cells/FOV (e) after 0‐, 4‐, 7‐, and 14‐day hypoxia. Results are expressed as the mean ± SEM (*n* = 4–6 mice/group). *p < 0.05, **p < 0.01, ***p < 0.001. Note that microglia in the aged brain are morphologically more active, express higher levels of Mac‐1 and show higher rates of proliferation at all time‐points. In addition, while young microglia show a mute response to hypoxia, those in aged brain exhibit a strong response.

Based on our recent findings that microglia play an important vasculo‐protective role during hypoxic exposure (Halder & Milner, 2019, 2020), an alternative possibility accounting for increased age‐related vascular leak is that microglial vasculo‐protection declines with age. In this regard, two points are worth considering. First, recent studies have shown that microglia in the aged brain are more activated or “primed,” than in the young brain (Godbout et al., 2005; Mosher & Wyss‐Coray, 2014; Streit et al., 2004). Second, microglia are historically regarded as a “double‐edged sword,” performing protective functions at low levels of activation, but contributing to disease pathogenesis when more strongly or persistently activated (Carson, 2002; Hanisch & Kettenmann, 2007). To investigate the idea that aged microglia may confer less vasculo‐protection, we first examined the influence of age and CMH on microglial activation by performing Mac‐1 IF. As shown in Figure 3a, most microglia in young normoxic brains displayed the ramified morphology (small cell body with long thin processes) typical of resting microglia, and in young mice, CMH had no discernible impact on microglial morphology, except for those microglia closely associated with vascular leaks, which displayed the typical activated morphology (large cell body and short thicker process extensions as shown in Figure S9). However, in the aged brain, we observed two key differences. First, even under normoxic conditions, microglia displayed a much more activated morphology. Second, CMH dramatically increased microglial activation in all brain areas in aged mice, even where no obvious vascular leak had occurred. Quantification of the number of morphologically activated microglia confirmed that CMH had little impact on microglial activation in young mice, while in contrast, microglia in aged mice were more activated under normoxic conditions (*p* < 0.001) and more strongly activated after CMH (*p* < 0.001, Figure 3c). Quantification of Mac‐1 signal per field of view (FOV) confirmed that microglia in aged brain were much more activated than in young brain (*p* < 0.01 under normoxic conditions and *p* < 0.001 during CMH, Figure 3d). Iba‐1 staining of aged brains under normoxic and hypoxic conditions confirmed similar changes in microglial activation (Figure S10). In addition, while microglia in young brain showed very little proliferation (Mac‐1+/Ki67+ dual‐positive cells) in response to CMH, microglia in aged brain showed a strong proliferation response to CMH (as denoted by arrows in Figure 3b and quantified in Figure 3e).

**FIGURE 4:**
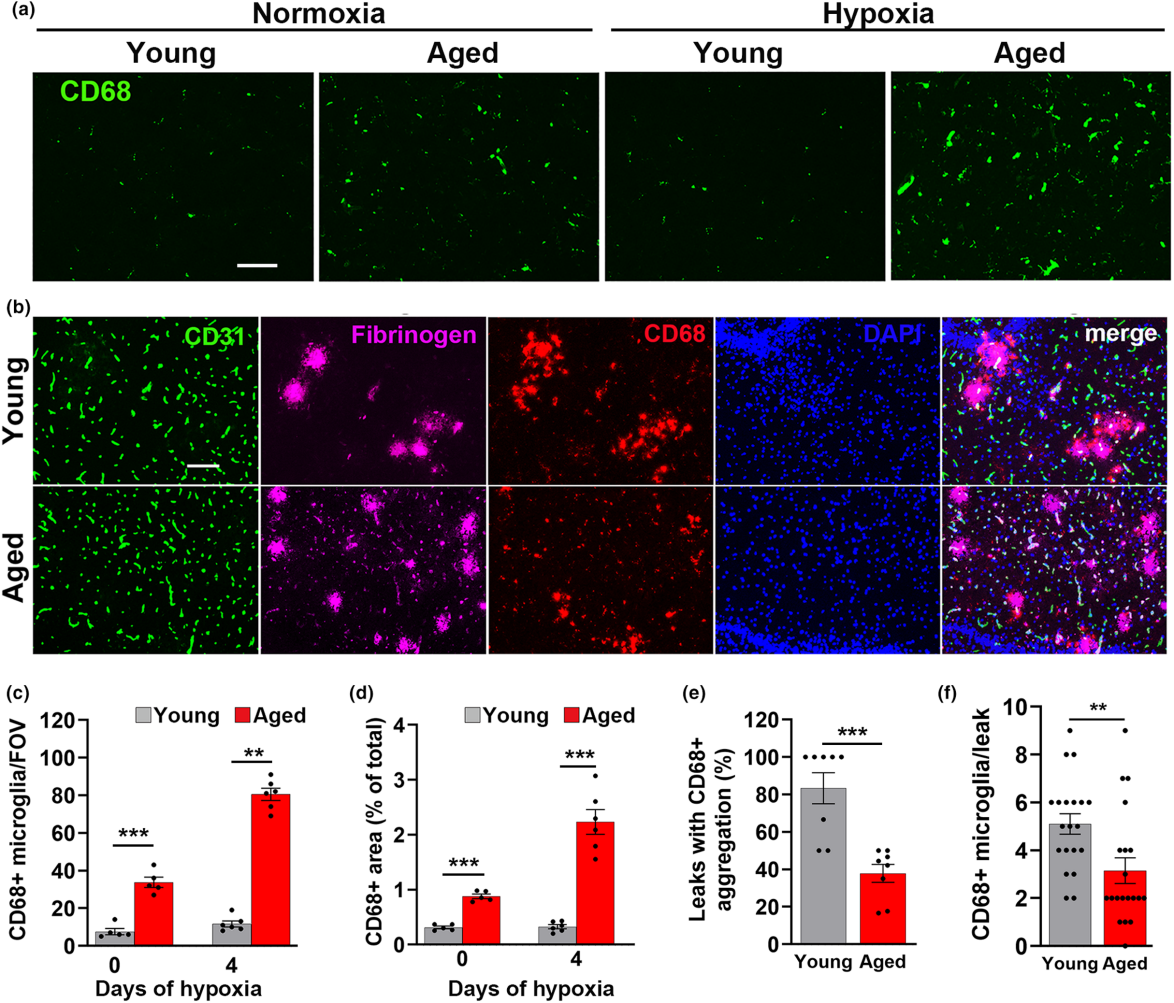
Microglia in aged brain show higher levels of CD68 expression but reduced aggregation around leaky blood vessels. Frozen brain sections taken from young and aged mice exposed to normoxia or hypoxia (8% O_2_) for 4 days were stained for CD68 (AlexaFluor‐488) (a) or CD31 (AlexaFluor‐488), fibrinogen (Cy‐5), CD68 (Cy‐3), and DAPI (blue). (b). Images were captured in the midbrain. Scale bars = 100 μm. Quantification of the number of CD68^+^ cells/FOV (c), total CD68 area/FOV (d), number of vascular leaks with aggregation of CD68^+^ cells (e), or number of CD68^+^ microglial cells per fibrinogen+ area (f) after 0‐ or 4‐day hypoxia. Results are expressed as the mean ± SEM (*n* = 5–6 mice/group). ** *p* <0.01, ***p < 0.001. Note that microglia in aged brain express higher levels of CD68 both under normoxic and hypoxic conditions but show a markedly reduced aggregation around fibrinogen+ vascular leaks.

In an alternative method, we also analyzed microglial expression of CD68, a lysosomal marker of microglial priming (Walker & Lue, 2015), specifically in brain areas not showing obvious vascular leak, in order to gain insight into how background CD68 levels differ with age (Figure 4). This showed that while the number of CD68^+^ cells was relatively low in young normoxic brains and little affected by CMH, CD68^+^ number was much higher in the aged normoxic brain (*p* < 0.001) and increased yet again in the aged hypoxic brain (*p* < 0.001; see Figure 4a and quantified in Figure 4c). Quantification of total CD68^+^ signal confirmed these findings (Figure 4d). In our recent study of young brain, we demonstrated that fibrinogen‐positive vascular leaks were almost always associated with marked accumulation of microglia displaying the hypertrophic activated phenotype (Halder & Milner, 2020). To examine the impact of age, we performed CD31/fibrinogen/CD68 triple‐IF on young and aged brains (Figure 4b). This revealed a marked difference in the way that microglia respond to vascular leak at the different ages. Whereas in young brain, the vast majority of fibrinogen+ vascular leaks were surrounded by aggregates of CD68^+^ activated microglia, in the aged brain many vascular leaks were not (Figure 4b) (83.3 ± 12.7% in young midbrain vs. 37.8 ± 7.4% in aged midbrain, *p* < 0.001). Analysis in the olfactory bulb showed a similar pattern (95.0 ± 5.1% in young vs. 48.1 ± 12.5% in aged brain, *p* < 0.001). Consistent with these findings, the number of CD68^+^ microglial cells per fibrinogen+ area was also reduced in the aged brain (5.1 ± 0.42 cells in young vs. 3.15 ± 0.52 cells in aged brain, *p* < 0.01, Figure 4f). This was particularly surprising considering that aged brains contain a much higher density of CD68^+^ cells (Figure 4a,c). Together, these data present an interesting paradox. On the one hand, they show that microglia in the aged brain are much more activated than in young mice, consistent with previous reports (Godbout et al., 2005; Mosher & Wyss‐Coray, 2014; Streit et al., 2004), and they also mount a stronger activation response to CMH than young microglia. However, despite this increased level of activation, microglia in the aged brain show a strikingly diminished ability to aggregate around leaky blood vessels.

### 2.5 Microglial depletion in aged mice results in greater hypoxia‐induced cerebrovascular leak

**FIGURE 5:**
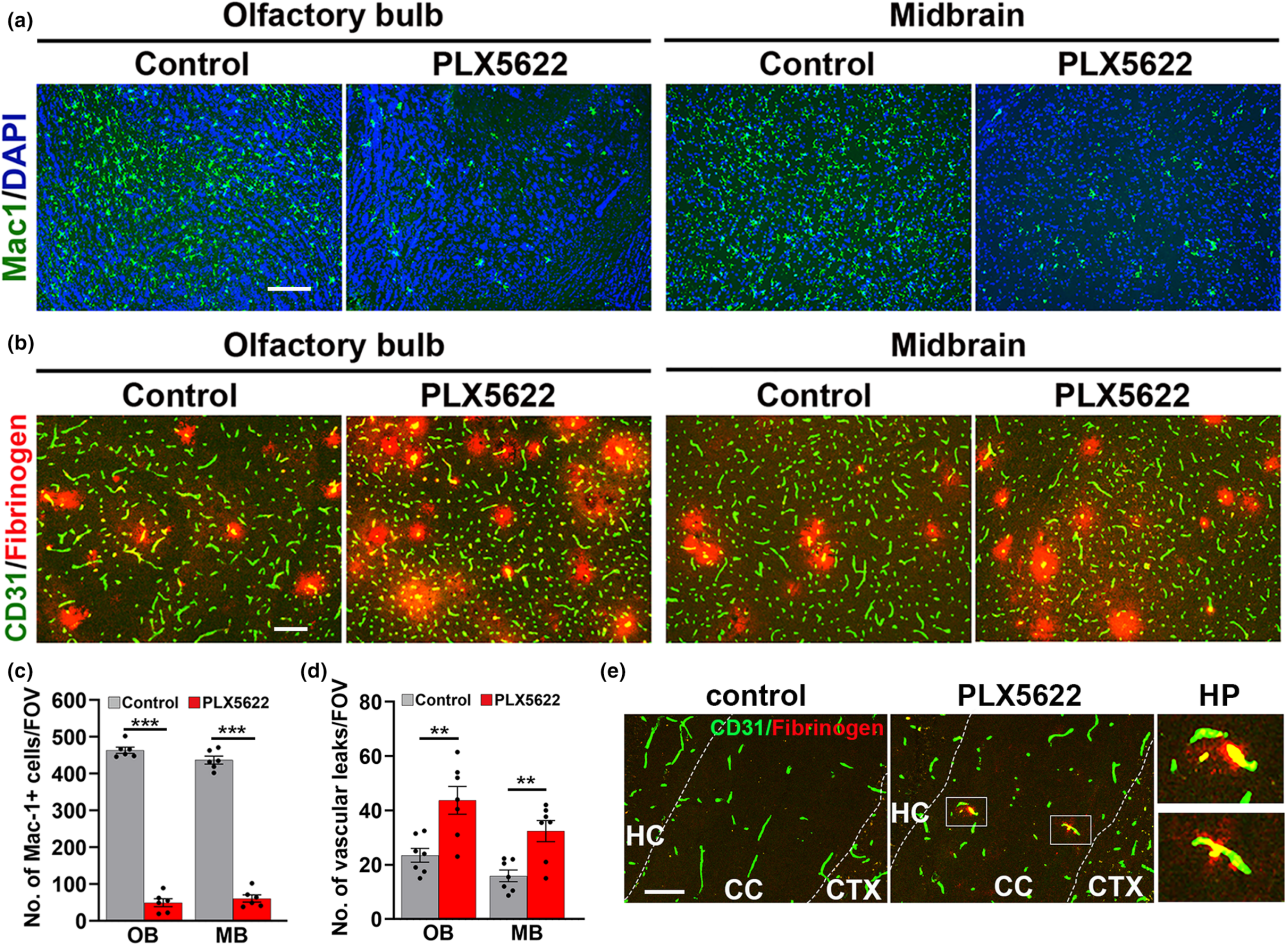
Microglial depletion in aged mice results in greater CMH‐induced cerebrovascular leak. (a) Frozen brain sections taken from aged mice fed normal chow or PLX5622‐containing chow and maintained under normoxic conditions for 21 days were stained for the microglial marker Mac‐1 (AlexaFluor‐488) and DAPI (blue). Scale bar = 200 μm. (b) Images of the olfactory bulb and midbrain taken from aged mice fed normal chow or PLX5622‐containing chow for 21 days before being maintained under hypoxic conditions for 4 days were stained for CD31 (AlexaFluor‐488) and fibrinogen (Cy‐3). Scale bar = 200 μm. (c,d) Quantification of microglial depletion after 21‐day PLX5622 in the olfactory bulb (OB) and midbrain (MB) (c) or the number of vascular leaks/FOV in the olfactory bulb (OB) and midbrain (MB) in aged mice fed normal chow or PLX5622‐containing chow and maintained under hypoxic conditions for 4 days (d). All results are expressed as the mean ± SEM (*n* = 6–7 mice/group). **p < 0.01. ***p < 0.001. Note that 21‐day PLX5622 reduced microglial density in both brain regions to less than 15% of untreated controls and that both brain regions in PLX5622‐treated mice showed a much higher number of vascular leaks. (e) CD31/fibrinogen dual‐IF images of the hippocampus (HC)/corpus callosum (CC)/cerebral cortex (CTX) regions taken from control chow or PLX5622‐treated mice maintained under normoxic control conditions. Scale bar = 100 μm. Note the presence of vascular leaks within the corpus callosum of normoxic PLX5622‐treated mice but not control mice.

Recently, we demonstrated that pharmacological depletion of microglia in young mice leads to greater hypoxia‐induced cerebrovascular leak, demonstrating that microglia play an important vasculo‐protective role (Halder & Milner, 2020). As our current data show that microglia in aged mice are more activated but paradoxically, less efficient, at mediating vasculo‐protection, this suggests that the overly activated microglia in the aged brain may have deviated from vasculo‐protective toward a vasculo‐destructive phenotype. If this is true, then removing microglia should result in less vascular leak. To investigate this possibility, we depleted microglia from aged mice using chow containing PLX5622, an inhibitor of colony stimulating factor 1 receptor (CSF‐1R), a well‐established pharmacological approach of depleting microglia (Elmore et al., 2018, 2014), and then examined how microglial depletion impacted hypoxia‐induced cerebrovascular leak. As shown in Figure 5a,c, aged mice treated with 1200 ppm (1200 mg drug per kg chow) PLX5622 for 21 days demonstrated >85% reduction in the number of brain microglia compared to untreated mice in all regions examined, including the midbrain and olfactory bulb (*p* < 0.001). Aged mice were pre‐treated with PLX5622 for 21 days, then exposed to CMH for 4 days with PLX5622 maintained throughout, and the impact on CMH‐induced vascular leak then examined. As shown in Figure 5b,d, this revealed that similar to our findings in young brain, microglial depletion in the aged brain resulted in an increased number of hypoxia‐induced vascular leaks (increased more than 2‐fold, *p* < 0.01) both in the olfactory bulb and the midbrain. Most interestingly, in aged mice treated with PLX5622, even under normoxic conditions, we occasionally observed vascular leak in specific brain regions such as the corpus callosum (Figure 5e). This implies that in these regions of the aged brain, microglial vasculo‐protective function is required to maintain vascular integrity, even under non‐challenged conditions. To examine whether hypoxia‐induced extravascular fibrinogen leak was also associated with hemorrhage, we performed dual‐IF for fibrinogen and the platelet marker CD41 (GPIIb) as a marker of blood leak into the brain parenchyma. This revealed that extravascular fibrinogen leak was often, but not always, associated with hemorrhage (Figure S11), and notably, microglial depletion with PLX5622 increased the percentage of leaks that showed signs of hemorrhage.

### 2.6 Pharmacological attenuation of microglial activation reduces the number of CMH‐induced vascular leaks in aged mice

**FIGURE 6:**
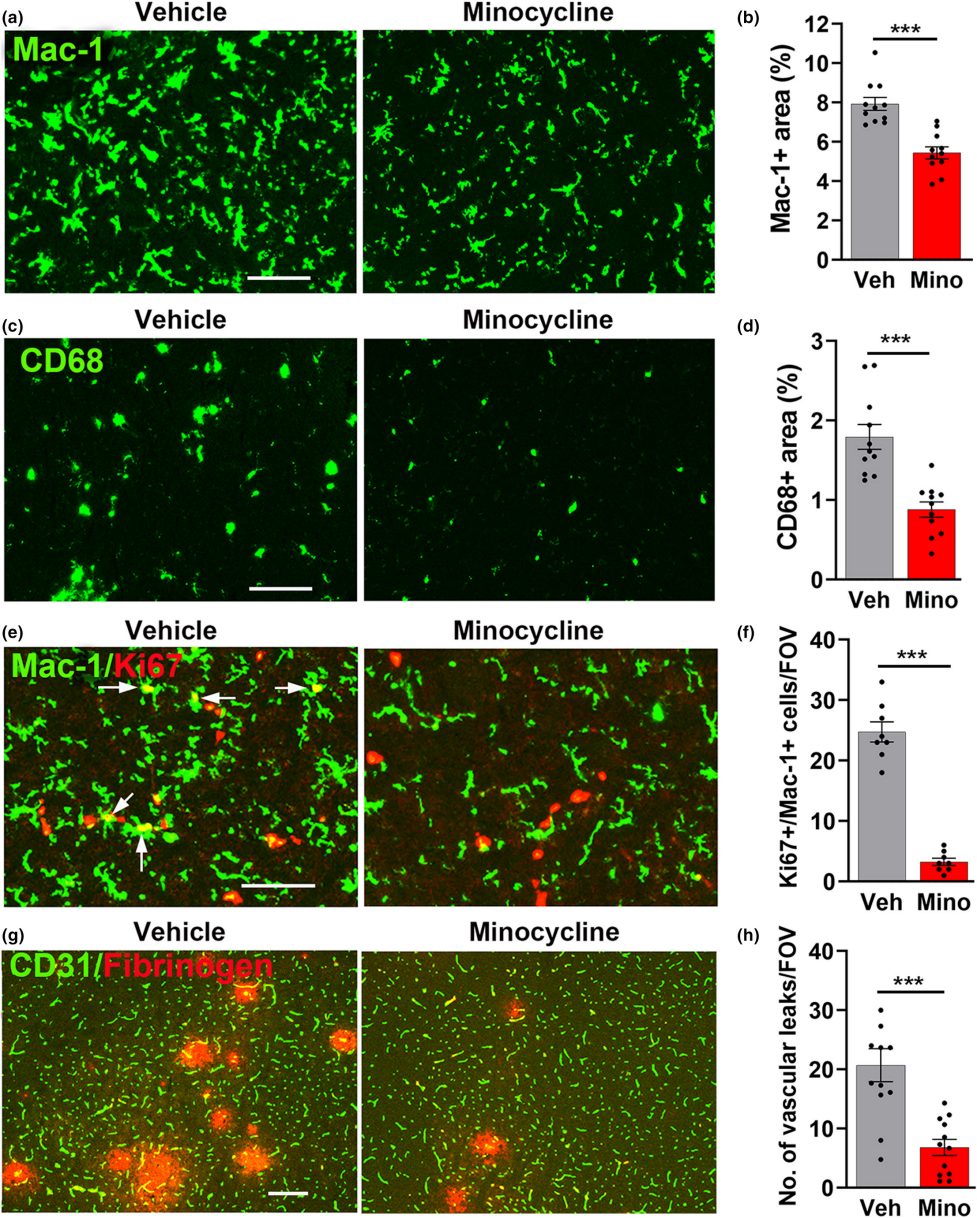
Minocycline reduces microglial activation in the aged brain, resulting in less vascular leak. Frozen brain sections from vehicle or minocycline‐treated aged mice exposed to hypoxia for 4 days were stained for Mac‐1 (AlexaFluor‐488) (a), CD68 (AlexaFluor‐488) (c), Mac‐1 (AlexaFluor‐488) and Ki67 (Cy‐3) (e), or CD31 (AlexaFluor‐488) and fibrinogen (Cy‐3) (g). Images were captured in the midbrain. Scale bars = 100 μm (a,c,e) or 200 μm (g). Proliferating microglia are denoted by arrows. Quantification of Mac‐1 area (b), CD68 area (d), number of Mac‐1+/Ki67+ cells/FOV (f), or number of vascular leaks (h). Results are expressed as the mean ± SEM (*n* = 8–12 mice/group). ***p < 0.001. Note that minocycline markedly reduced microglial activation markers and proliferation, resulting in fewer vascular leaks.

As microglial depletion leads to greater vascular leak in the aged brain, this demonstrates that microglia in the aged brain still play a net vasculo‐protective role. However, as aged microglia appear to be less effective in their vasculo‐protective capacity, this suggests that when microglia become too activated in the aged brain, this undermines their vasculo‐protective function. If this is true, then reducing microglial activation should lead to less vascular leak. To test this idea, we used minocycline, a well‐established method of reducing microglial activation (Manso et al., 2018; Zhao et al., 2007). Aged mice were exposed to CMH for 4 days in the absence (vehicle) or presence of minocycline (administered daily i.p. at 50 mg/kg). This showed that aged mice treated with minocycline showed significantly reduced levels of Mac‐1 (*p* < 0.001) and CD68 (*p* < 0.001) compared to vehicle controls (Figure 6a–d). Minocycline also strongly inhibited CMH‐induced microglial proliferation in aged mice (*p* < 0.001; Figure 6e,f). Most importantly, compared with vehicle controls, aged mice treated with minocycline showed significantly reduced numbers of CMH‐induced vascular leaks in all areas of the brain, including the midbrain (*p* < 0.001, shown in Figure 6g,h) and the olfactory bulb (*p* < 0.001, Figure S12). In parallel, minocycline attenuated the CMH‐induced loss of endothelial tight junction proteins ZO‐1 and occludin (denoted by arrows in Figure S13), that is typically observed in leaky blood vessels (*p* < 0.001). Interestingly, when we treated young mice with the same dose of minocycline, this also significantly reduced the number of CMH‐induced vascular leaks in all areas of the brain, including the midbrain and the olfactory bulb (Figure S14). Taken together, these results demonstrate that minocycline attenuates hypoxia‐induced cerebrovascular leak, both in aged and young mice.

## 3 DISCUSSION

These studies demonstrate that hypoxia‐induced cerebrovascular leak is greatly amplified (5‐fold to 10‐fold) in aged mice due to a combination of delayed vascular remodeling and reduced microglial vasculo‐protection. Furthermore, while overly activated microglia in the aged brain are less effective at promoting vascular integrity, this protective function can be partially restored by reducing microglial activation with minocycline, suggesting therapeutic potential for the hypoxia‐predisposed elderly population.

### 3.1 Hypoxia‐induced vascular leak is greatly enhanced in the aged brain

The findings presented here are consistent with previous studies demonstrating age‐associated decline in BBB integrity (Farrall & Wardlaw, 2009; Senatorov et al., 2019). While this is not totally surprising considering general age‐related functional decline in all systems, what is surprising is the magnitude of these changes. For instance, in the two regions showing the greatest extent of hypoxia‐induced vascular leak, the olfactory bulb and the midbrain, the number of vascular leaks in aged mice is 5‐fold to 10‐fold that seen in young mice, and this difference is observed in almost all other regions. Interestingly, in brain regions showing very few leaks in young mice, such as the cerebral cortex, striatum, and thalamus, this ratio becomes even greater than 10‐fold, demonstrating that in the aged brain, hypoxia has an even greater detrimental impact on cognitive regions not affected in young mice. Because relative hypoxia is common to many medical conditions, including sleep apnea, asthma (both of which can occur early in life), as well as COPD and age‐related cardiac and cerebrovascular insufficiency, many of which are established risk factors for cognitive decline (Leng et al., 2017; Russ et al., 2020; Yaffe et al., 2011; Yohannes et al., 2017), these findings have major clinical implications for a large fraction of the population.

### 3.2 Regional vulnerability

A significant finding from this study was that two specific brain regions showed high susceptibility to hypoxia‐induced vascular leaks: the olfactory bulb and the midbrain. This raises two important questions: (i) why are these regions leakier than others, and (ii) does this have any clinical implications? Interestingly, previous studies have highlighted the olfactory bulb as a hotspot of vascular breakdown, both under hypoxic conditions and in animal models of viral and malarial infection (Hoffmann et al., 2016; Winkler et al., 2015; Zhao et al., 2014). In one of these studies, it was suggested that the trabeculated small capillaries of the olfactory bulb are uniquely sensitive to breakdown (Zhao et al., 2014). Another reason could be that because the olfactory bulb is one of the most dynamically plastic areas of the adult brain, with the ability to generate new neurons via the rostral migratory stream throughout life (Wilson et al., 2004), this is accompanied by an equally high level of vascular plasticity. In support of this idea, we showed recently that the olfactory bulb shows the highest rate of vascular remodeling in response to hypoxia (Halder & Milner, 2020). Interestingly, we found that increased vulnerability to hypoxia‐induced vascular breakdown in the olfactory bulb correlated with marked impairment of olfactory function, demonstrating that vascular leak has important implications for brain function. This regional vulnerability may also hold clinical relevance because several studies have suggested that loss of smell (anosmia) may be an important early predictor of cognitive decline and dementia (Adams et al., 2018; Growdon et al., 2015). Aside from the olfactory bulb, the number of vascular leaks in the midbrain was also very striking. While some leaks occurred within the substantia nigra (SN), many were also more widespread in the midbrain, with the red nucleus (RN) particularly affected. As both the SN and RN structures are involved with motor control and coordination, this raises the possibility that hypoxic insults may predispose to the pathogenesis of movement disorders, including Parkinson's disease.

### 3.3 Delayed vascular remodeling in the aged

Our studies demonstrate that although aged mice are still capable of launching a vascular remodeling response to CMH, this is markedly delayed compared to young mice. Indeed, the endothelial proliferation response in aged brain diminished at a much faster rate and this was accompanied by a delay in vessel maturation, indicated by extended MECA‐32 expression, thus predisposing to longer periods of vascular vulnerability and greater leak. This concept is supported by our recent work showing that transgenic mice lacking endothelial expression of the angiogenic integrin α5β1 (α5‐EC‐KO) show greater vascular leak in a neuroinflammatory model (Kant et al., 2019). These observations are also in keeping with previous studies describing loss of angiogenic potential with age (Benderro & LaManna, 2011; Black et al., 1989). In particular, Benderro et al described delayed hypoxia‐induced angiogenesis in the aged mouse cortex, which they attributed to age‐related attenuation of HIF‐1α and VEGF expression levels (Benderro & LaManna, 2011). Alternatively, the extended MECA‐32 expression in aged mice might be because less efficient angiogenesis fails to correct tissue hypoxia as it does in young animals; thus, the vessels remain leaky for longer. It is important to acknowledge that despite the delay in vessel maturation and subsequent enhanced vascular leak in aged mice, in the long term, remodeling blood vessels in the hypoxic aged brain retain the inherent ability to repair any disruptions, as illustrated by the reduced number of vascular leaks remaining after 14‐day CMH compared to earlier timepoints. However, in the face of continued and increasingly severe episodes of hypoxia in aged patients that accumulate over many years, it seems likely that these protective mechanisms may eventually be overwhelmed.

### 3.4 Microglial vasculo‐protection declines with age

**FIGURE 7:**
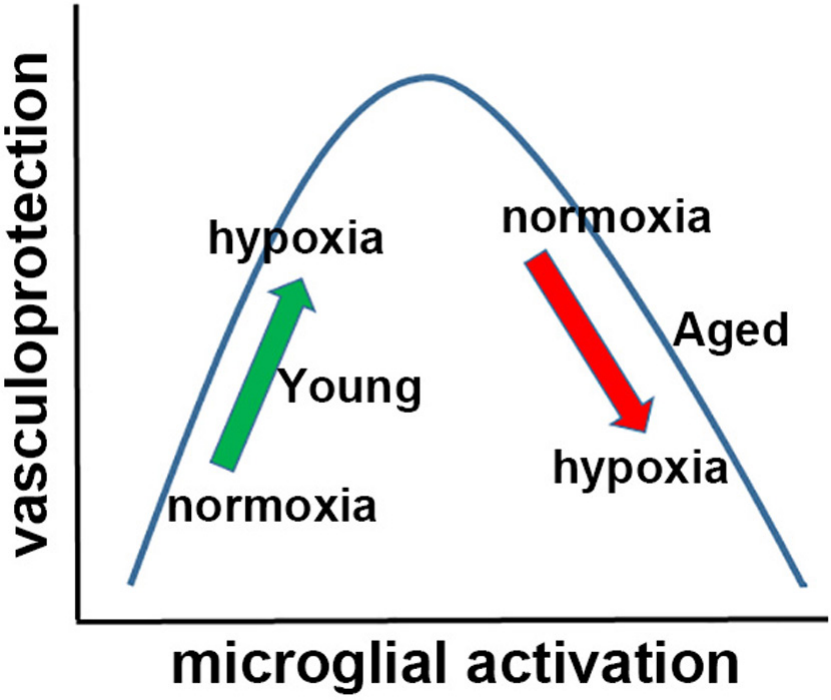
Model proposing a biphasic relationship between microglial activation vasculo‐protective function. According to this model, microglia in young normoxic mice (green arrow) are resting but upon hypoxic‐induced vascular leak, they become more activated and display enhanced vasculo‐protective function. In contrast, microglia in aged normoxic mice (red arrow) occupy a higher baseline activation state, so that when stimulated by hypoxia, they become overly activated and less vasculo‐protective.

Building on our recent findings that in young mice, microglia play an important vasculo‐protective role in response to hypoxia (Halder & Milner, 2019, 2020), we wondered if microglia in the aged brain may be less activated by hypoxia. In fact, we saw the opposite was true because under normoxic conditions, microglia in the aged brain expressed higher levels of activation markers and looked morphologically more activated compared to those in young brain, and unlike the young brain, in the aged brain, hypoxia enhanced microglial activation status. Together, these data present an interesting paradox; despite microglia in the aged brain being much more activated than in young mice, they show a strikingly diminished ability to aggregate around leaky blood vessels. Based on these findings, we wondered if microglia in the aged brain had switched from playing a vasculo‐protective role to more of a vasculo‐destructive one, but this idea was disproved by our finding that microglial depletion in aged brain exacerbated vascular leak, confirming that microglia in the aged brain still play an overall vasculo‐protective role. However, there remained the possibility that as higher microglial activation state correlated with worse vascular leak in aged mice, attenuating microglial activation to a level seen in young mice, might improve the vasculo‐protective function of aged microglia. To this end, recent studies have employed a reprogramming strategy whereby all aged microglia are removed by PLX5622, to be replaced by endogenous microglia having a “younger” phenotype (Elmore et al., 2018; O'Neil et al., 2018). In one particular study, with obvious implications for our work, this approach was shown to reverse age‐related cognitive decline (Elmore et al., 2018). Here, we took a more direct approach by using minocycline, a well‐established method of attenuating microglial activation (Manso et al., 2018; Zhao et al., 2007). This demonstrated that in aged mice, minocycline strongly reduced microglial activation responses, correlating with significantly fewer hypoxia‐induced vascular leaks, as well as diminished loss of tight junction protein expression. Based on these data, we propose a model (Figure 7) in which microglial vasculo‐protective function displays a biphasic relationship with activation state. According to this model, microglia in young normoxic mice (green arrow) are resting but upon hypoxic‐induced vascular leak, they become more activated and display enhanced vasculo‐protective function. In contrast, microglia in aged normoxic mice (red arrow) occupy a higher baseline activation state, so that when stimulated by hypoxia, they become overly activated and less vasculo‐protective. The effect of minocycline in aged mice is consistent with this model because minocycline attenuation of microglial activation left‐shifted microglia back into the protective range. Intriguingly, as minocycline also reduced hypoxia‐induced vascular leak in young mice, this suggests that even in young mice, there is a “sweet point” at which microglia confer maximal vasculo‐protection, and that microglial activation over and above this point is detrimental to the vasculo‐protective process. The outstanding question now becomes: why are overly activated microglia less effective at vasculo‐protection? One possibility, based on the biphasic relationship that exists between the strength of cell adhesion and speed of migration (DiMilla et al., 1993; Palecek et al., 1997), is that highly activated microglia in the aged brain are more strongly attached to the surrounding substrates by way of increased expression of cell adhesion molecules including integrins (Kloss et al., 2001; Milner & Campbell, 2003), and are thus less free to migrate at optimal speed to disrupted blood vessels. A second possibility is that because in aged brain, the leak of fibrinogen and other chemoattractants is far more extensive and diffuse, this may greatly reduce the chemoattractant gradient, resulting in flattened microglial aggregation responses. Third, it is also possible that overly activated microglia could release pro‐inflammatory soluble factors such as cytokines or matrix metalloproteinases (MMPs) that negatively impact vascular integrity (Boroujerdi et al., 2015; Rosenberg, 2002). In future studies, we plan to address some of these possibilities.

## 4 EXPERIMENTAL PROCEDURES

### 4.1 Animals

The studies described were reviewed and approved by the Explora Biolabs Institutional Animal Care and Use Committee at San Diego Biomedical Research Institute (SDBRI). Wild‐type female C57BL6/J mice obtained from Jackson Laboratories were maintained under pathogen‐free conditions in the closed breeding colony of SDBRI.

### 4.2 Chronic Hypoxia Model

Female C57BL6/J mice, 8–10 weeks (young) and 20 months (aged), were housed 4 to a cage, and placed into a hypoxic chamber (Biospherix) maintained at 8% O_2_ for periods up to 14 days. Littermate control mice were kept in the same room under similar conditions except that they were kept at ambient sea‐level oxygen levels (normoxia, approximately 21% O_2_ at sea‐level) for the duration of the experiment. Every few days, the chamber was briefly opened for cage cleaning and food and water replacement as needed.

### 4.3 Buried food test

Olfactory function was evaluated using the well‐established buried food test, which evaluates the ability of mice to locate a food source hidden beneath a layer of bedding, as previously described (Yang & Crawley, 2009). The purpose of this test is to measure an animal's ability to smell odors and use these cues to forage and find hidden food. The protocol involves a 3‐day process involving odor familiarization and tasting the food source on Day 1, overnight fasting on Day 2, and testing on Day 3. On test day, mice were allowed to acclimatize for 5 min to a clean cage containing 3 cm think bedding. The mouse was temporarily removed while a Teddy Graham cookie (Nabisco) was buried beneath 1 cm of bedding in a random corner of the cage. The mouse was then reintroduced into the cage, and the time taken for the mouse to find the buried cookie was measured in seconds up to a maximum of 10 min (with 600 s as the maximum score).

### 4.4 Elimination of microglia

PLX5622 was provided by Plexxikon under Material Transfer Agreement and formulated in AIN‐76A standard chow by Research Diets at a dose of 1200 p.p.m. (1200 mg PLX5622 in 1 kg chow). In microglial depletion experiments, aged mice were fed a PLX5622 diet for 21 days prior to being placed in the hypoxic chamber to deplete microglia before they were exposed to hypoxic conditions. Once in the hypoxic chamber, these mice were then maintained on the PLX5622 diet for 4 days. Consistent with the findings of others, in these studies we found no obvious behavioral alterations, weight loss or signs of illness in mice fed a PLX5622 diet for 3 weeks (Elmore et al., 2018, 2014).

### 4.5 Attenuation of microglial activation

To evaluate the impact of attenuated microglial activation, aged mice exposed to CMH for 4 days received daily intraperitoneal (i.p.) injections of minocycline (50 mg/kg; Sigma‐Aldrich) or vehicle (PBS).

### 4.6 Immunohistochemistry and antibodies

Immunohistochemistry was performed on 10 μm frozen sections of cold phosphate buffer saline (PBS) perfused tissues as described previously (Boroujerdi et al., 2015). For Iba‐1 staining, following PBS perfusion, brains were fixed in 4% paraformaldehyde for 24 hrs then immersed in 25% sucrose for 24 hrs before sections cut. Rat monoclonal antibodies from BD Pharmingen reactive for the following antigens were used in this study: CD31 (clone MEC13.3; 1:500), CD41 (clone MWReg30: 1:100), MECA‐32 (1:100), Mac‐1 (clone M1/70; 1:50), CD68 (clone FA‐11; 1:2000), and VCAM‐1 (clone 429; 1:100). The hamster anti‐CD31 (clone 2H8; 1:500) and rabbit anti‐NeuN (clone EPR12763; 1:2000) monoclonals were obtained from Abcam and mouse monoclonal anti‐TH‐1 (clone LNC1; 1:300) from Millipore‐Sigma. Rabbit antibodies reactive for the following proteins were also used: Ki67 (1:4000 from Novus Biologicals), fibrinogen (1:1500 from Millipore), AQP4 (1:10,000 from Alomone Labs), Iba‐1 (1:1000 from DAKO), and occludin and ZO‐1 (1:1500 from Invitrogen). The sheep anti‐fibrinogen antibody (1:3000) was obtained from Bio‐Rad and the goat anti‐CD206 antibody (1:500) from R&D. Secondary antibodies used (all at 1:500) included Cy3‐conjugated anti‐rabbit, anti‐rat, anti‐goat and anti‐mouse and Cy5‐conjugated anti‐rabbit from Jackson Immunoresearch, and Alexa Fluor 488‐conjugated anti‐rat, anti‐hamster, anti‐sheep, anti‐goat, and anti‐rabbit from Invitrogen.

### 4.7 Image analysis

Images were taken using a 5×, 10×, or 20× objective on a Zeiss Imager M1.m fluorescent microscope. For each antigen in all analyses, images of at least three randomly selected areas were taken at 10× or 20× magnification per tissue section and three sections per brain analyzed to calculate the mean for each animal (*n* = 4–12 mice per group). For each antigen in each experiment, exposure time was set to convey the maximum amount of information without saturating the image and was maintained constant for each antigen across the different experimental groups. The number of vascular leaks or MECA‐32+ or VCAM‐1+ vessels per field of view (FOV) was quantified by capturing images and performing manual counts of the number of vessels showing extravascular leaked fibrinogen, MECA‐32 or VCAM‐1, respectively. The number of activated microglia was quantified by performing manual counts of the number of CD68^+^ cells or by morphological criteria of Mac‐1 or Iba‐1 staining (large cell body and short process extensions) per FOV. Total number of Mac‐1+ microglia were quantified by performing manual counts. Total Mac‐1, Iba‐1, or CD68^+^ area fluorescent signal per FOV was measured and analyzed using NIH Image J software. Endothelial and microglial proliferation was quantified by counting the number of CD31/Ki67 or Mac‐1/Ki67 dual‐positive cells per FOV, respectively. The number of vessels lacking expression of AQP4 or the tight junction proteins ZO‐1 and occludin, or the number of fibrinogen+ leaky vessels showing CD68^+^ microglial accumulation or CD41+ platelets was quantified by capturing images and performing manual counts. Each experiment was performed with 4–12 different animals per condition, and the results expressed as the mean ± SEM. Statistical significance was assessed using one‐way analysis of variance (anova) followed by Tukey's multiple comparison post hoc test, in which *p* < 0.05 was defined as statistically significant.

## AUTHOR CONTRIBUTIONS

SKH performed the mouse hypoxia studies and the histological analysis. SKH and RM analyzed the data. RM conceived of the study and drafted the manuscript. Both authors read and approved the final manuscript.

## CONFLICT OF INTEREST

The authors report no competing interests.

## Supporting information


Figures S1–S14

## Data Availability

The data that support the findings of this study are available from the corresponding author upon reasonable request.
