# Supplementary material for: Exaggerated hypoxic vascular breakdown in aged brain due to reduced microglial vasculo‐protection
Source: Aging Cell. 2022 Sep 21;21(11):e13720. doi: 10.1111/acel.13720 (PMC9649604; doi:10.1111/acel.13720)
Supplement: Supplementary file 1 — Figures S1–S14 [file ACEL-21-e13720-s001.docx]

Supplementary Information for:

**Exaggerated hypoxic vascular breakdown in aged brain due to reduced microglial vasculo-protection**

Sebok K. Halder and Richard Milner

Email: rmilner@sdbri.org

**This PDF file includes:**

Supplementary Figures S1 to S14

**Figure S1**

**
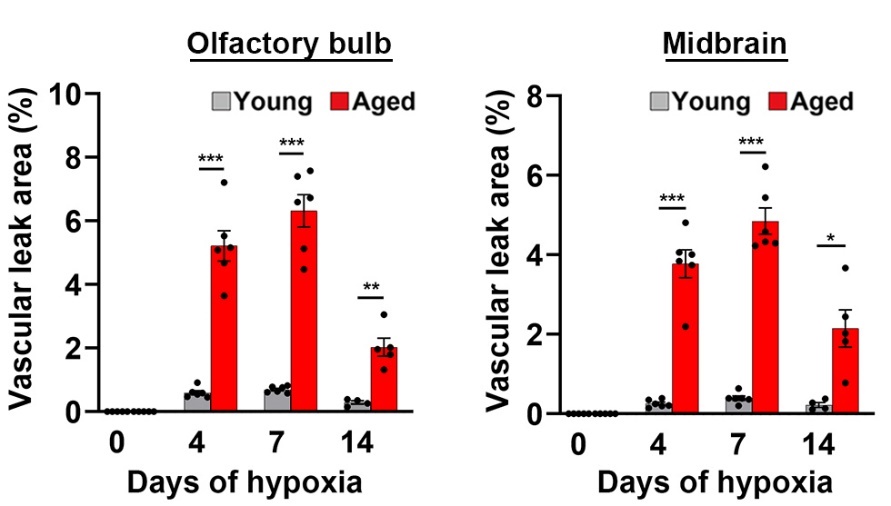
**

**Figure S1. Aged mice show greater hypoxia-induced vascular leak.** Quantification of the area of vascular leak in the olfactory bulb and midbrain after 0, 4, 7 and 14-days hypoxia. All results are expressed as the mean ± SEM % area (n = 4-6 mice/group). * p < 0.05, ** p < 0.01, *** p < 0.001 vs. normoxic conditions. Note that in both the olfactory bulb and midbrain, the area of vascular leak was much greater in aged brain and that this peaked after 7 days hypoxia but declined by day 14.

**Figure S2**


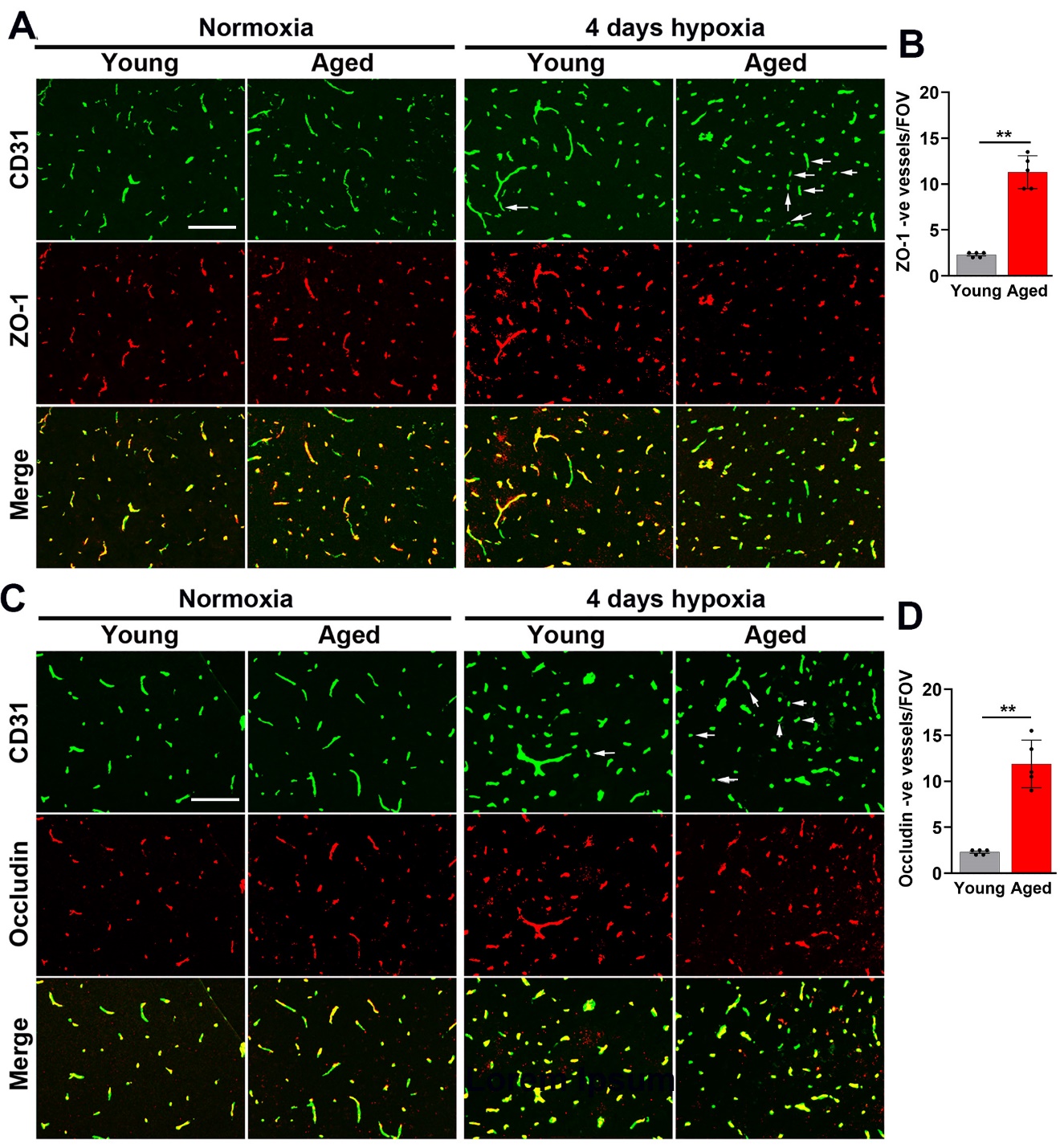


**Figure S2. CMH-induced vascular loss of tight junction proteins is much greater in aged brain.** **A.** Frozen brain sections taken from young (8-10 weeks) or aged (20 months) mice exposed to normoxia or 4 days hypoxia (8% O_2_) were stained for CD31 (AlexaFluor-488) and ZO-1 (Cy-3) in panel **A** or CD31 (AlexaFluor-488) and occludin (Cy-3) in panel **C**. Images were captured in the midbrain. Scale bar = 100 μm. Quantification of the number of ZO-1 (**B**) or occludin (**D**)-negative blood vessels/FOV. Results are expressed as the mean ± SEM (n = 5 mice/group). ** p < 0.01. Note that in aged brains, the number of tight junction-deficient blood vessels (see arrows in A and C) was much higher.

**Figure S3**


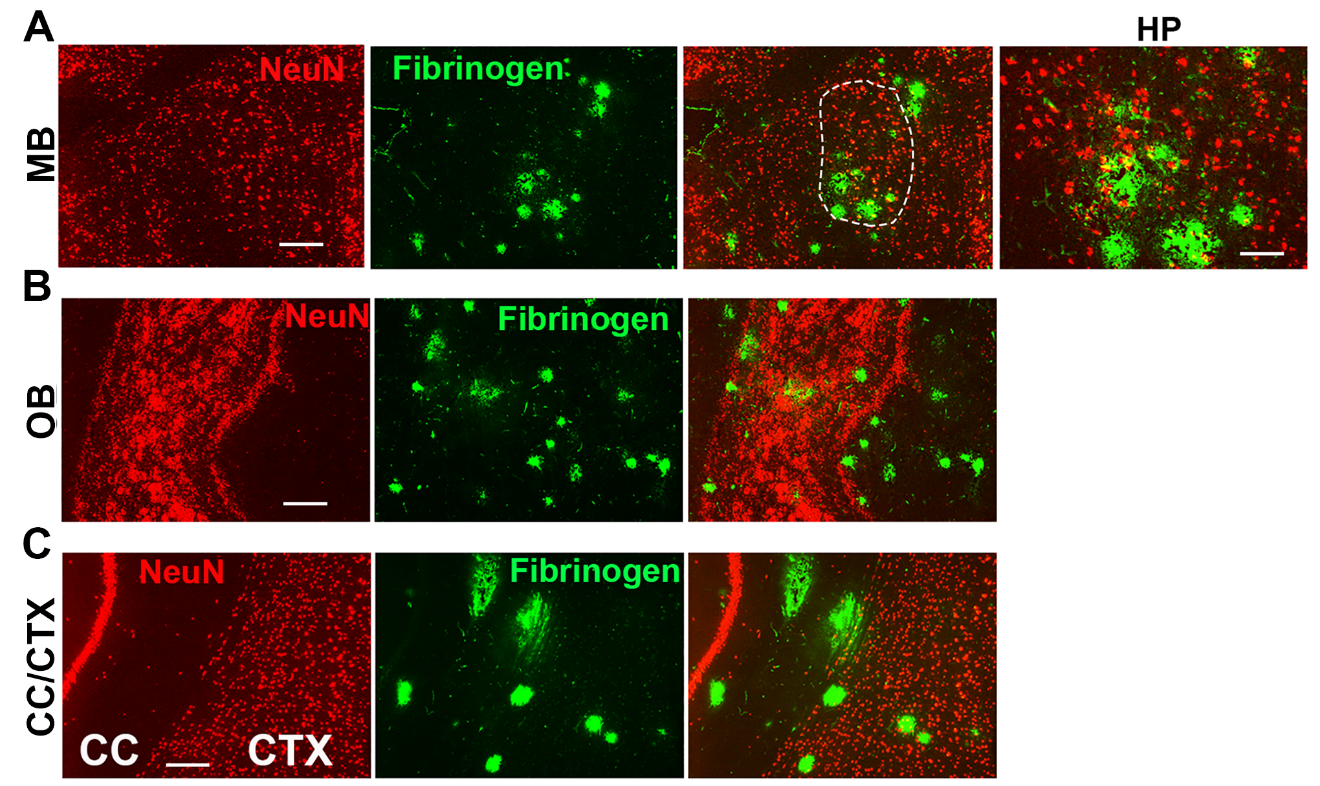


**Figure S3. Closer analysis of region-specific vascular leak in aged mice exposed to hypoxia.** **A-C.** Frozen brain sections taken from aged mice exposed to 7 days hypoxia (8% O_2_) were dual-labelled for NeuN (Cy-3) and fibrinogen (AlexaFluor-488). Images were captured in three different brain regions: midbrain (MB), olfactory bulb (OB), and corpus callosum (CC)/cerebral cortex (CTX)). Scale bar = 200 μm (except for high power (HP) image on the right in A, where scale bar = 100 μm). Note that hypoxia-induced vascular leak occurred in the red nucleus in the midbrain (area outlined in Panel **A**), both in NeuN+ grey matter and NeuN-negative white matter in the olfactory bulb (**B**), and in the white matter of the corpus callosum and grey matter of the cerebral cortex (**C**).

**Figure S4**


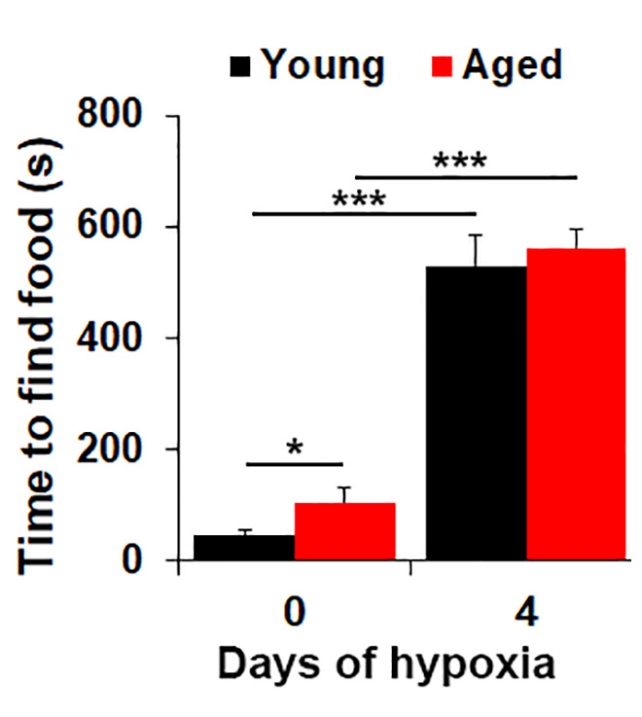


**Figure S4. Exposure to CMH impairs olfactory function.** Evaluation of how long it takes for young and aged mice to find buried food following 4 days of normoxic or hypoxic conditions. Results are expressed as the mean ± SEM seconds (n = 6 mice/group). * p < 0.05, *** p < 0.001. Note that under normoxic conditions, aged mice took twice as long as young mice to find the hidden food, but mice exposed to 4 days CMH (both young and aged) took much longer to find the hidden food.

**Figure S5**

**
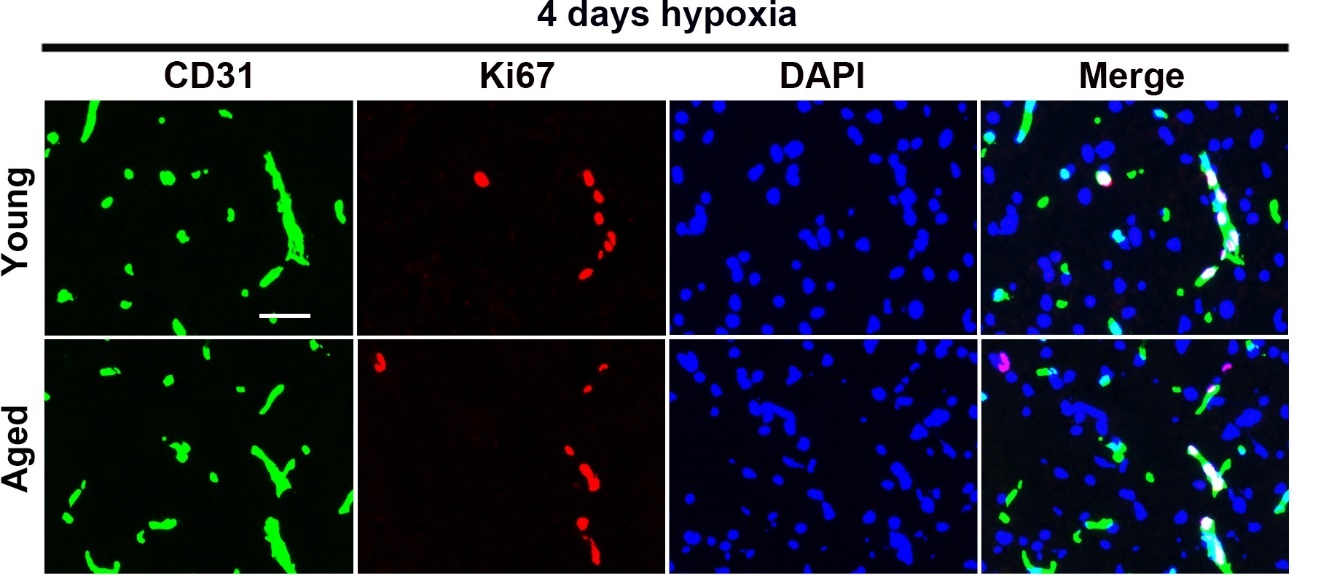
**

**Figure S5. CMH induces endothelial proliferation in both young and aged brains.** Frozen brain sections from young or aged mice exposed to hypoxia for 4 days were stained for CD31 (AlexaFluor-488), Ki67 (Cy-3) and DAPI (blue). Images were captured in the midbrain. Scale bar = 50 μm.

**Figure S6**

**
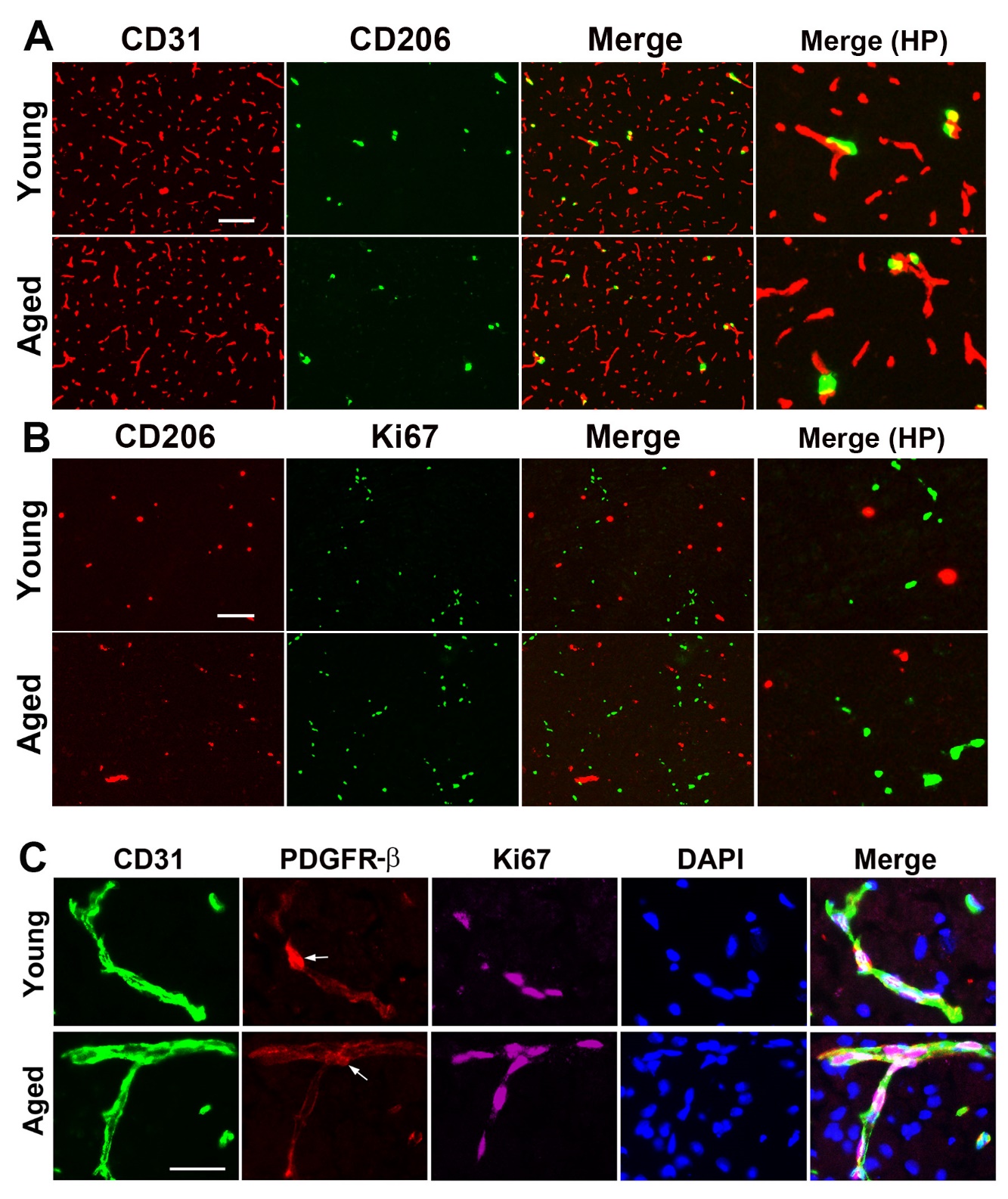
**

**Figure S6. CMH induces a strong proliferation response in endothelial cells but not in perivascular macrophages or pericytes.** Frozen brain sections taken from young (8-10 weeks) or aged (20 months) mice exposed to 4 days hypoxia (8% O_2_) were stained for CD31 (Cy-3) and the perivascular macrophage marker CD206 (AlexaFluor-488) (**A),** CD206 (Cy-3) and Ki67 (AlexaFluor-488) (**B**), or CD31 (AlexaFluor-488), PDGFRβ (Cy-3), and Ki67 (Cy-5) **C**. Images were captured in the midbrain. Scale bar = 100 μm. Note that CD206+ perivascular macrophages closely associated with CD31+ blood vessels (A) but they never associated with Ki67+ nuclei (B), indicating that they rarely proliferated. PDGFRβ+ pericyte extensions ran the length of blood vessels, thus these extensions often co-localized with CD31+Ki67+ cells, but pericyte cell bodies (arrows) were only rarely Ki67+ (C), indicating that most proliferating cells were endothelial cells.

**Figure S7**


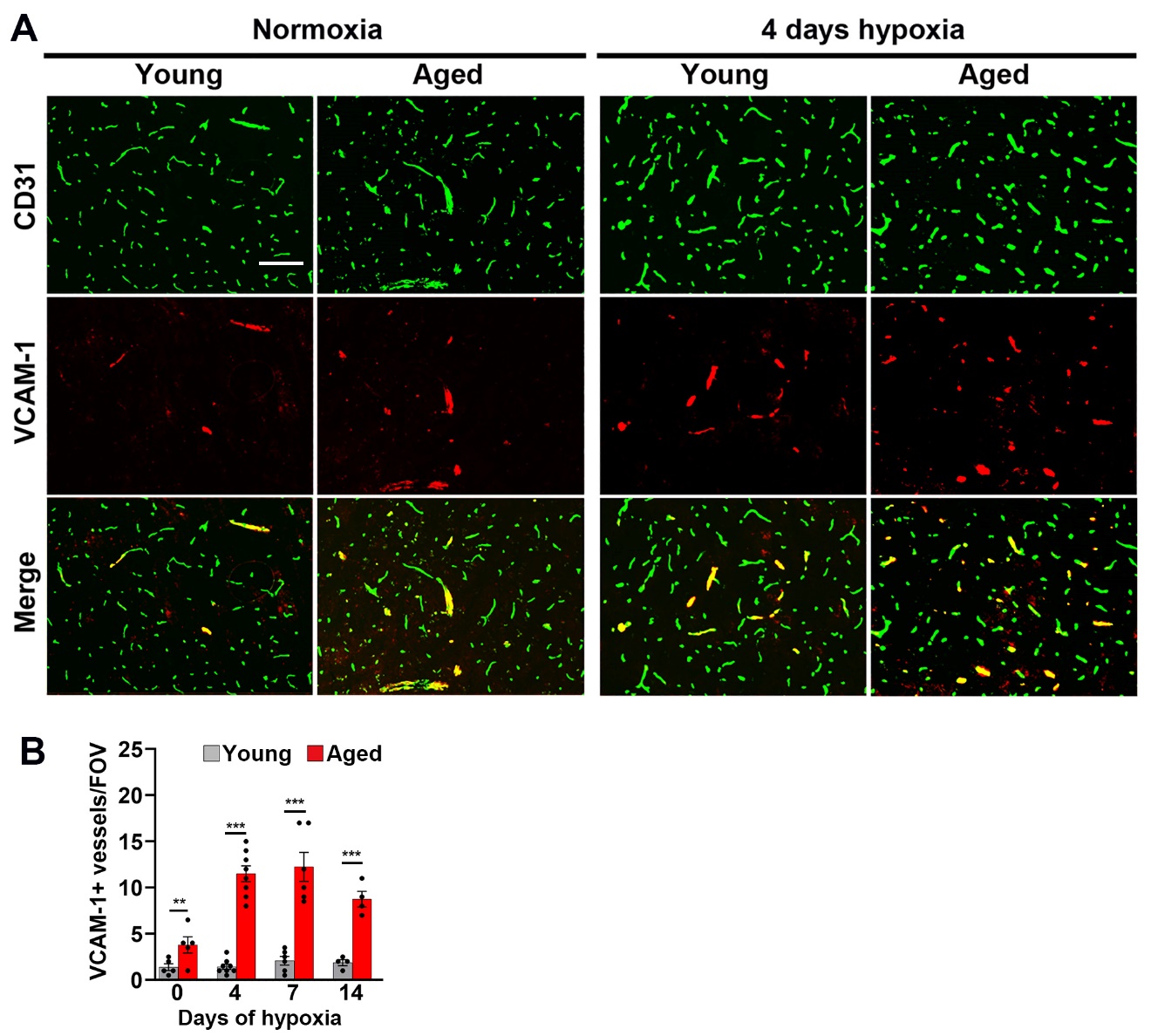


**Figure S7. Increased VCAM-1 expression on cerebral blood vessels in aged mice.** **A**. CD31/VCAM-1 dual-IF of frozen brain sections (midbrain shown) taken from young or aged mice exposed to normoxia or 4 days hypoxia (8% O_2_). Scale bar = 100 μm. **B**. Quantification of the number of VCAM-1+ vessels/FOV after 0, 4, 7, and 14 days hypoxia. Results are expressed as the mean ± SEM (n = 4-8 mice/group). ** p < 0.01, *** p < 0.001. Note that aged brains contained a greater number of VCAM-1+ vessels at all time-points.

**Figure S8**


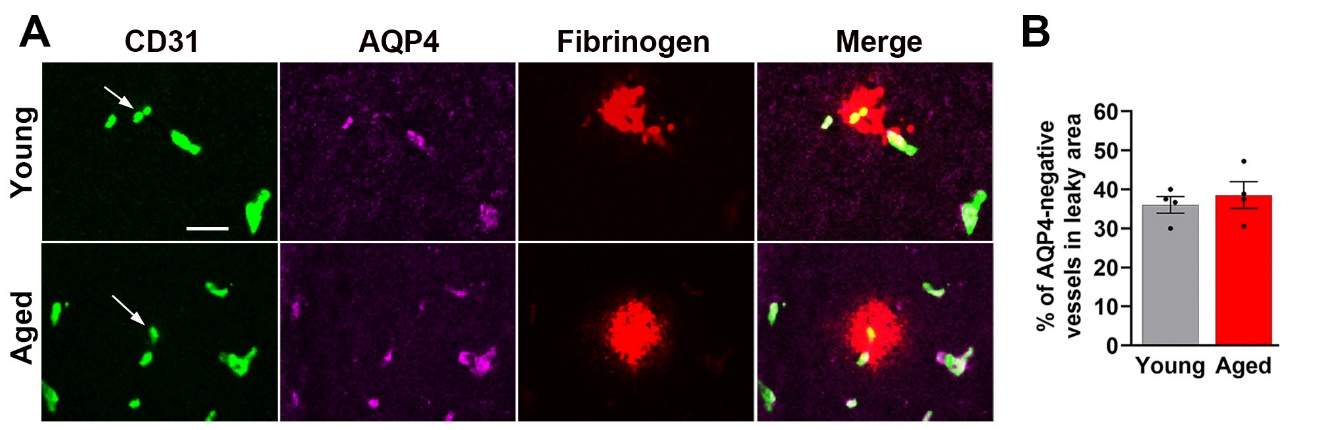


**Figure S8. Loss of astrocyte-vascular coupling in areas of extravascular fibrinogen leak.** **A.** Frozen brain sections taken from mice exposed to hypoxia for 4 days were stained for CD31 (AlexaFluor-488), AQP4 (Cy-5) and fibrinogen (Cy-3). Arrows denote AQP4-negative vessels. Images were captured in the midbrain. Scale bar = 25 μm. **B.** Quantification of the % of AQP4-negative vessels in areas of vascular leak in the midbrain. Results are expressed as the mean ± SEM (n = 4 mice/group). Note that vessels in leaky areas lost AQP4 expression in both young and aged brain, and that the % of vessels within leaky vessels that showed loss of AQP4 was not noticeably different between the two ages.

**Figure S9**


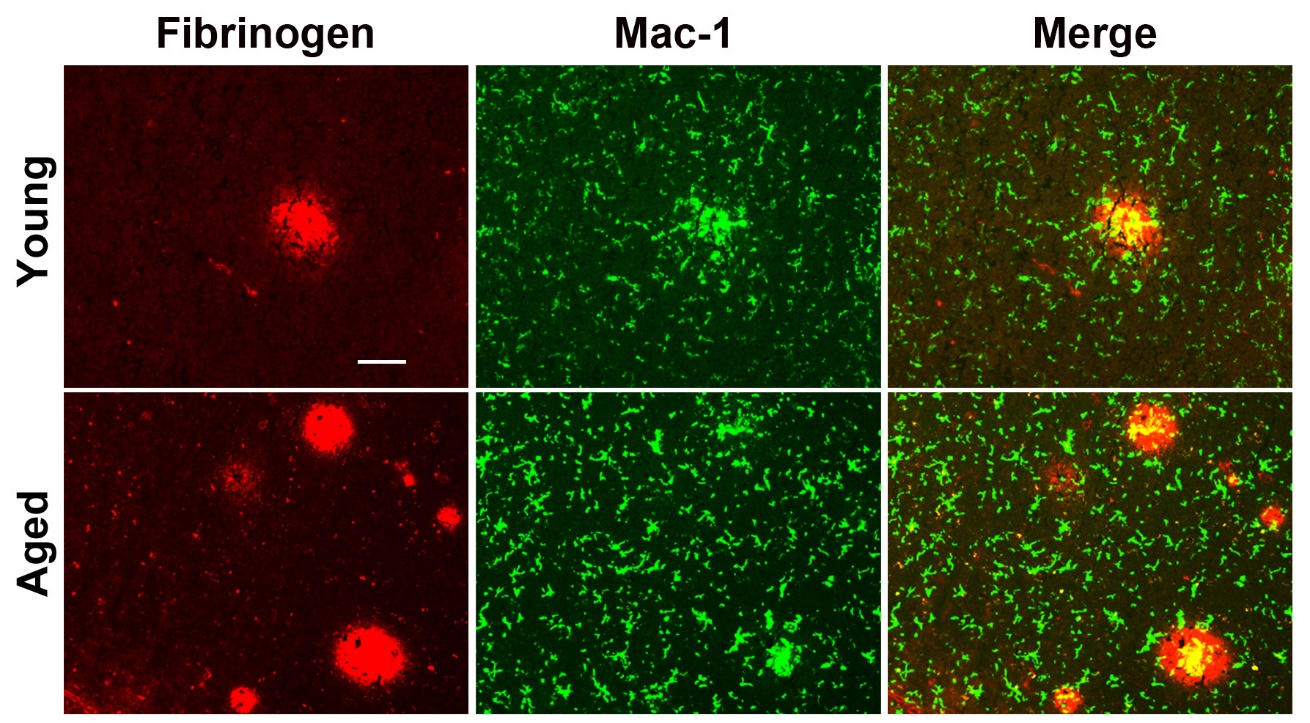


**Figure S9. Microglia surrounding vascular leaks are strongly activated.** Frozen brain sections taken from young (8-10 weeks) or aged (20 months) mice exposed to hypoxia (8% O_2_) for 4 days were stained for fibrinogen (Cy-3) and Mac-1 (AlexaFluor-488). Images were captured in the midbrain. Scale bar = 100 μm. Note that the vast majority of microglia in the young hypoxic brain displayed the ramified morphology (small cell body with long thin processes) typical of resting microglia, except for those microglia closely associated with vascular leaks, which displayed the typical activated morphology (large cell body and short thicker process extensions). In the aged hypoxic brain, microglia surrounding vascular leaks were also strongly activated but microglia in non-leaky areas were also more activated than in young brain.

**Figure S10**


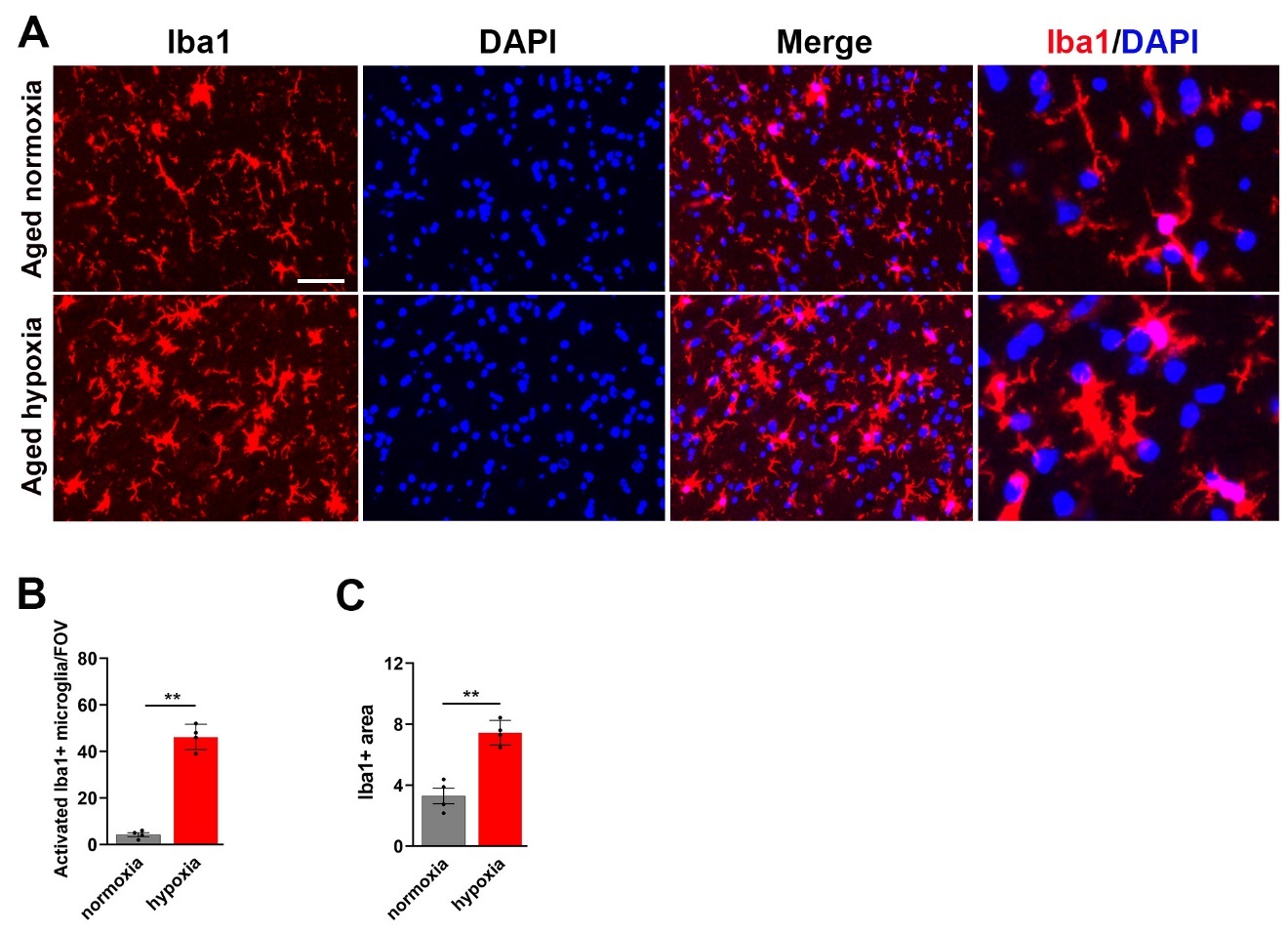


**Figure S10. Microglia in aged brain are strongly activated by CMH.** **A.** Frozen brain sections taken from aged (20 months) mice exposed to normoxia or hypoxia (8% O_2_) for 4 days were stained for Iba-1 (Cy-3) and DAPI (blue). Images were captured in the midbrain. Scale bar = 50 μm. **B.** Quantification of the number of morphologically activated microglia/FOV. C. Total Iba-1 area/FOV. Results are expressed as the mean ± SEM (n = 4 mice/group). ** p < 0.01. Note that microglia in the aged brain are strongly activated by CMH.

**Figure S11**


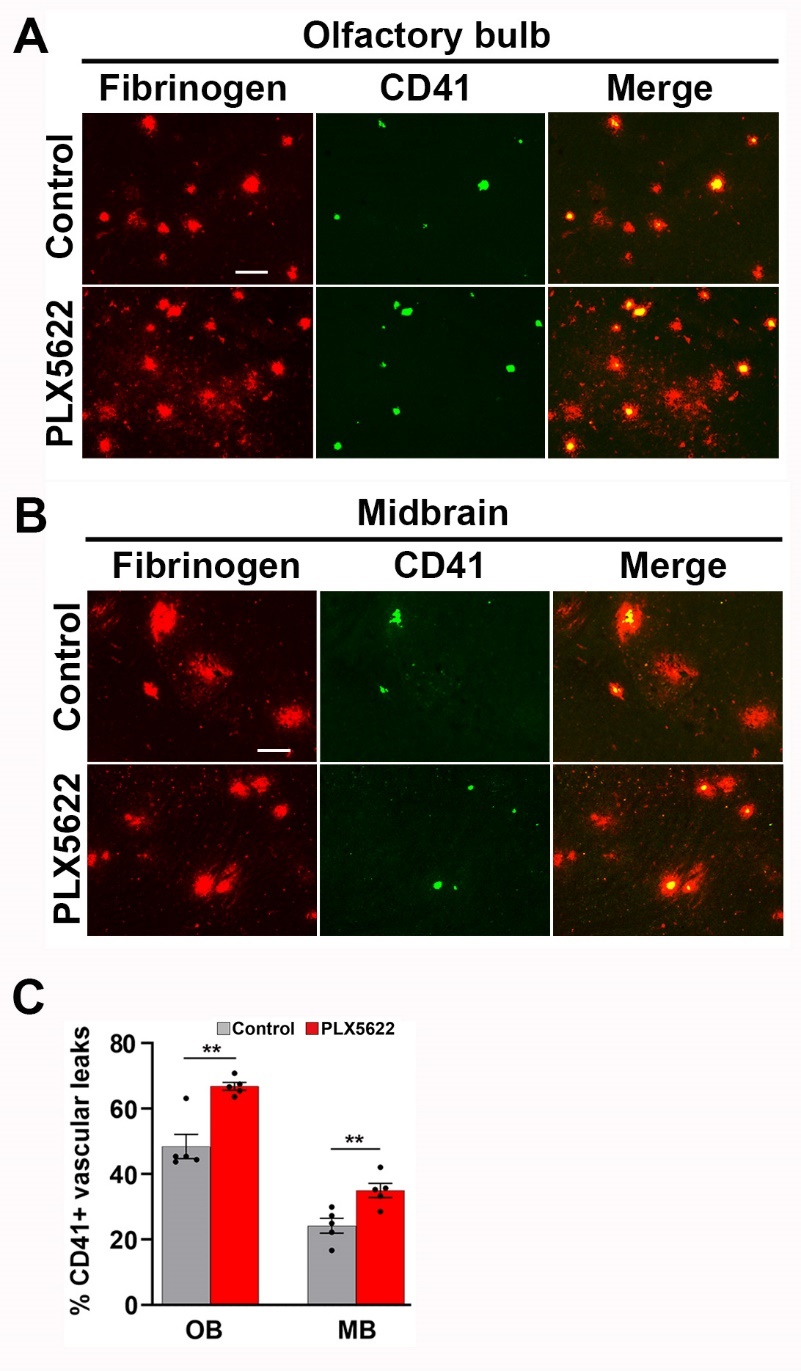


**Figure S11. Extravascular fibrinogen leak is associated with hemorrhage in the aged brain.** Frozen brain sections from aged mice exposed to hypoxia for 4 days were stained for fibrinogen (Cy-3) and CD41 (AlexaFluor-488). Images display the olfactory bulb **(A)** and the midbrain **(B).** Scale bars = 200 μm. Note the association between extravascular fibrinogen leak and extravascular deposition of platelets expressing CD41. **C.** Quantification of the % of vascular leaks in the olfactory bulb and midbrain that were associated with CD41 expressing platelets. Results are expressed as the mean ± SEM (n = 5 mice/group). Note that this association was increased in microglia-depleted mice. ** p < 0.01

**Figure S12**


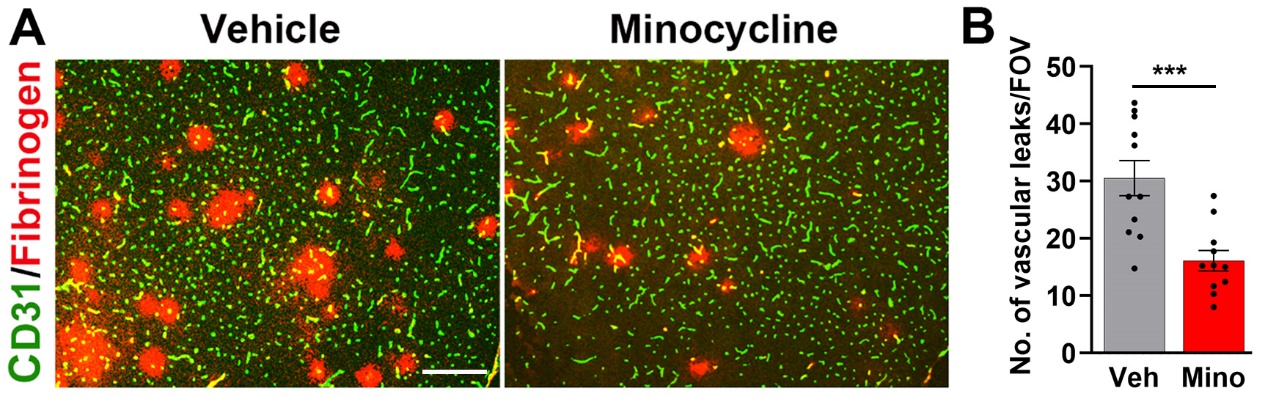


**Figure S12. Minocycline attenuates CMH-induced vascular leak in the olfactory bulb.** **A**. Frozen brain sections taken from vehicle or minocycline-treated aged mice exposed to hypoxia for 4 days were stained for CD31 (AlexaFluor-488) and fibrinogen (Cy-3). Scale bar = 200 μm. B. Quantification of the number of vascular leaks/FOV. Results are expressed as the mean ± SEM (n = 11-12 mice/group). *** p < 0.001. Note that minocycline markedly reduced CMH-induced vascular leak in the olfactory bulb.

**Figure S13**


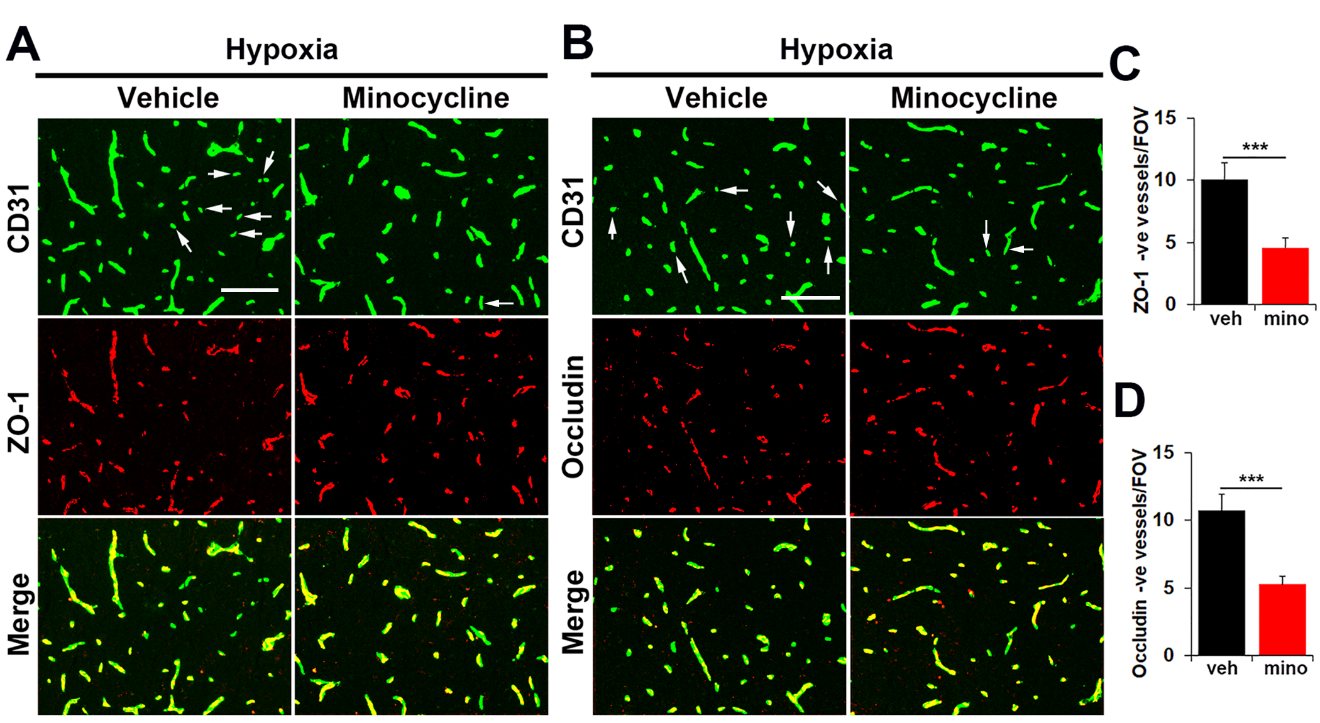


**Figure S13. Minocycline reduces CMH-induced loss of tight junction proteins in aged brain.** Frozen brain sections taken from vehicle or minocycline-treated aged mice exposed to hypoxia for 4 days were stained for CD31 (AlexaFluor-488) and ZO-1 (Cy-3) in panel **A** or CD31 (AlexaFluor-488) and occludin (Cy-3) in panel **B**. Images were captured in the midbrain. Scale bar = 100 μm. Quantification of the number of ZO-1 (**C**) or occludin (**D**)-negative blood vessels/FOV in the midbrain. Results are expressed as the mean ± SEM (n = 6 mice/group). *** p < 0.001. Note that minocycline markedly reduced CMH-induced vascular loss of ZO-1 and occludin (see arrows in A and B).

**Figure S14**


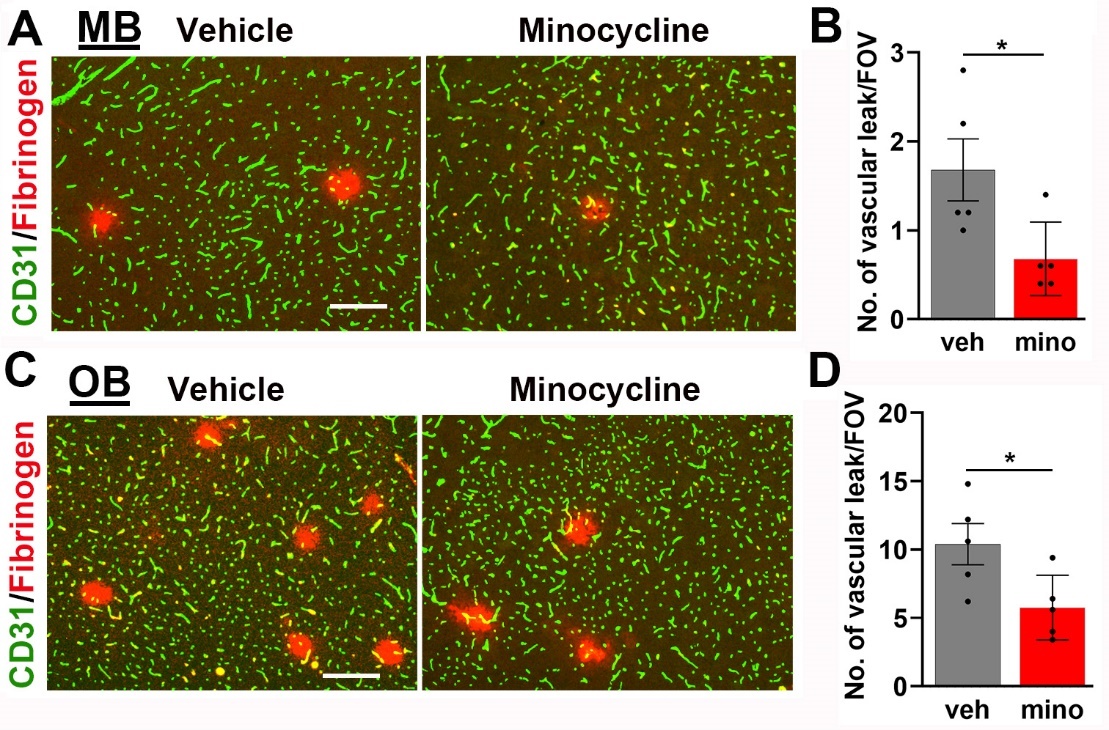


**Figure S14. Minocycline reduces vascular leak in the young brain.** Frozen brain sections from vehicle or minocycline-treated young mice exposed to hypoxia for 4 days were stained for CD31 (AlexaFluor-488) and fibrinogen (Cy-3). Images display the midbrain **(A)** and olfactory bulb **(C).** Scale bars = 200 μm Quantification of the number of vascular leaks in the midbrain **(B)** and olfactory bulb **(D).** Results are expressed as the mean ± SEM (n = 5 mice/group). * p < 0.05. Note that minocycline reduced vascular leak in young brain.
